# Genome‐scale Metabolic Modeling Guided *Escherichia coli* Engineering for *De Novo* Biosynthesis of Chrysanthemic Acid

**DOI:** 10.1002/advs.202512736

**Published:** 2025-11-21

**Authors:** Jiangpeng Yu, Kelin Cheng, Shenyang Qu, Jie Wang, Xun Wang, Cheng Zhao, Wei Li

**Affiliations:** ^1^ Shenzhen Branch Guangdong Laboratory of Lingnan Modern Agriculture Key Laboratory of Synthetic Biology Ministry of Agriculture and Rural Affairs Agricultural Genomics Institute at Shenzhen Chinese Academy of Agricultural Sciences Shenzhen 518124 China; ^2^ College of Plant & Technology Huazhong Agricultural University Wuhan 430070 China; ^3^ National Key Laboratory of Agricultural Microbiology College of Life Science and Technology Huazhong Agricultural University Wuhan 430070 China

**Keywords:** cell factory, chrysanthemic acid, debranching strategy, E. coli, Genome‐scale metabolic model

## Abstract

Chrysanthemic acid is an unconventional monoterpene moiety of the natural pesticide pyrethrins with notable anti‐insect activity, making its industrial biosynthesis a promising avenue for sustainable agriculture. Here, an *E. coli* cell factory is designed and build for highly efficient chrysanthemic acid production guided by Genome‐scale metabolic models (GEM). The biosynthetic pathway is reconstructed and simulated the metabolic changes caused by exogenous modules are simulated. A key metabolic branch point catalyzed by ispA is identified by this model, and inhibiting its expression using synthetic small RNA redirected the metabolic flux, resulting in the titers of precursor chrysanthemol and chrysanthemic acid increasing by 162% and 59%, respectively. The effect of the expression level of downstream dehydrogenases on chrysanthemic acid titer is also predicated using GEM, and the further optimization of copy number for dehydrogenase genes led to a notably 570% increase in chrysanthemic acid titer experimentally. By integrating the debranching strategy with copy number optimization, a record chrysanthemic acid titer 141.78 mg L^−1^ is achieved in a bioreactor. The work seamlessly integrated in silico modeling optimization with wet‐lab practices that significantly enhance target metabolite titer through metabolic network engineering, offering a new route for constructing efficient cell factories for natural bioproducts.

## Graphical abstract

Genome‐scale metabolic modeling is preceded to the engineered *E. coli* cell factory for the de novo biosynthesis of biopesticide chrysanthemic acid. The debranching strategy redirects metabolic flux, enhancing precursor and target compound production. This integrated in silico and in vivo approach culminates in a record‐high titer, establishing a powerful paradigm for future metabolic engineering to produce valuable natural products.

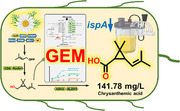

## 1 Introduction

Pyrethrins, a type of natural insecticide from pyrethrum (*Tanacetum cinerariifolium*), face development constraints due to costly plant extraction and complex chemical synthesis.^[^ ^1^ ^]^ Despite efforts in traditional plant breeding and agricultural optimization, these approaches haven't made pyrethrins an affordable agricultural insecticide. Chrysanthemic acid, one of pyrethrin's acid components, is also considered as a natural insecticide due to its anti‐insect activity.^[^ ^2^, ^3^ ^]^ Additionally, it serves as a crucial raw material for synthesizing pyrethroid insecticides like allethrin and deltamethrin.^[^ ^4^ ^]^ However, its complex stereochemistry makes chemical synthesis challenging, resulting in numerous by‐products and complicated purification steps.^[^ ^5^ ^]^

**Figure 1:**
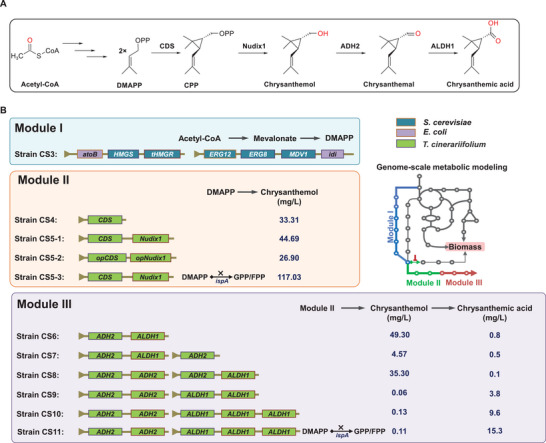
Construction of *E. coli* chrysanthemic acid cell factory modules and the titer of each module in this study. A) Simplified biosynthetic pathway of chrysanthemic acid from acetyl‐CoA. B) Module I: DMAPP supply module; Module II: Chrysanthemol biosynthesis module; Module III: Chrysanthemic acid biosynthesis module. *E. coli*: purple; *S. cerevisiae*: blue; *T. cinerariifolium*: green. *AtoB*, acetoacetyl‐CoA thiolase from *E. coli*; *HMGS*, HMG‐CoA synthase; *tHMGR*, truncated HMG‐CoA reductase; *ERG12*, mevalonate kinase; *ERG8*, phosphomevalonate kinase; *MVD1*, mevalonate pyrophosphate decarboxylase; *idi*, IPP isomerase from *E. coli*; *CDS*, chrysanthemyl diphosphate synthase, *Nudix1*, Chloroplast nudix_hydrolase; *ADH2*, alcohol dehydrogenase 2; *ALDH1*, Aldehyde dehydrogenase 1.

The chrysanthemic acid biosynthetic pathway has been elucidated in pyrethrum. Its molecular structure features a unique monoterpene backbone, formed by the head‐to‐middle condensation of two dimethylallyl diphosphate (DMAPP) units.^[^ ^6^ ^]^ Chrysanthemyl diphosphate synthase (CDS) condenses two DMAPP molecules from the plastid's methyl‐D‐erythritol phosphate (MEP) pathway to form chrysanthemyl diphosphate (CDP).^[^ ^6^ ^]^ Subsequently, phosphatases, including Nudix1, hydrolyzed CDP into chrysanthemol.^[^ ^7^ ^]^ ADH2 then oxidizes chrysanthemol to chrysanthemal, which ALDH1 further oxidizes to yield chrysanthemic acid (**Figure** **1**A).^[^ ^8^ ^]^

Several bioengineering studies for chrysanthemic acid have been performed using plant chassis, demonstrating the potential for heterologous production. Xu et al. successfully reconstituted the pathway in isoprenoid‐rich tomato fruit, introducing a novel concept for vegetable pest control, with a recorded titer of ≈1.09 µmol g^−1^ dry weight.^[^ ^9^ ^]^ Similarly, Hu et al. detected volatile chrysanthemol at ∼50 pmol h^−1^ g^−1^ FW and 1.1 mm by overexpressing *CDS* in *Chrysanthemum morifolium*, providing dual protection against aphids.^[^ ^3^ ^]^ Despite these advances, it's notable that no research has yet reported the production of chrysanthemic acid using microbial chassis.

Numerous successful instances have proved the effectiveness of *E. coli* for natural metabolite production, including terpenes biosynthesis. Martin et al. optimized the heterologous synthesis of artemisinin precursors, reaching 24 µg L^−1^; the *E. coli* MVA metabolic pathway they constructed serves as a basis for the synthesis of other terpenes.^[^ ^10^ ^]^ Ajikumar et al. modularized the taxadiene metabolic pathway in *E. coli*, resulting in the production of a titer of ≈1 g L^−1^.^[^ ^11^ ^]^ Park et al. reported the production of 432 mg L^−1^ of astaxanthin in *E. coli* by heterologous expression.^[^ ^12^ ^]^ Sun et al. employed genetic engineering and achieved an impressive yield of 3520 mg L^−1^ lycopene.^[^ ^13^ ^]^

The key to building microbial cell factories is engineering controlled and efficient biosynthesis systems, which requires iterative optimization of the cellular modules cooperation and the module adaptation to the microbial host, under the condition of balancing material and energy metabolism.^[^ ^14^ ^]^ Genome‐scale Metabolic Models (GEMs) have become a valuable tool in systems‐level metabolic engineering designs, offering comprehensive descriptions of the metabolic network.^[^ ^15^ ^]^ The model network encompasses knowledge in biochemistry and genomics, including precise chemical stoichiometry of metabolic reactions, chemical formulas, and charges of metabolites, along with associations among genes, proteins, and reactions.^[^ ^16^ ^]^ GEM enables the prediction of cellular phenotypes at the system level, guiding genetic manipulations to enhance growth and maximize target compound productivity.^[^ ^17^ ^]^

In this study, we enhanced chrysanthemol and chrysanthemic acid production in *Escherichia coli* via an open‐source, cost‐effective strategy. This involved introducing a DMAPP supply module and integrating both chrysanthemol and chrysanthemic acid modules. Leveraging transcriptomics and metabolomics data, we developed a GEM to simulate our modified strains. Using the machine‐learning tool UniKP, we screened DMAPP‐related branch reactions and, in combination with metabolic network modeling, identified ispA as the key branch point, which was subsequently validated experimentally. Guided by the model, we pinpointed the rate‐limiting step in chrysanthemic acid synthesis, boosting production by optimizing key gene copy numbers. Through iterative optimization, including module integration, we achieved flask titers exceeding 117.0 mg L^−1^ for chrysanthemol and 15.3 mg L^−1^ for chrysanthemic acid. Scaling to a 2L fermenter further increased chrysanthemic acid titer to 141.78 mg L^−1^, demonstrating the feasibility of large‐scale fermentation for future pyrethrin‐derived natural insecticide production.

## 2 Material and Methods

### 2.1 Strains, Plasmids and Chemical Reagents

We utilized *E. coli* Top10 and DH5α strains for gene cloning and plasmid construction. *E. coli* BL21 (DE3) was employed for protein expression and compound fermentation. Details of the plasmids and *E. coli* strains used can be found in Tables S1,S2 (Supporting Information), respectively.

Primers that were necessary for our experiments were synthesized by Sangon Biotech in China. We purified DNA and performed gel electrophoresis using kits from Macherey–Nagel.

Codon‐optimized genes for *E. coli* were synthesized by Sangon Biotech. For culturing *E. coli* Top10 and plasmid amplification, we used Luria–Bertani (LB) medium in 250 mL Erlenmeyer flasks. Antibiotics were added as needed, with the following concentrations: kanamycin sulfate (Kan, 50 µg mL^−1^), carbenicillin (Amp, 100 µg mL^−1^), chloramphenicol (Cm, 17 µg mL^−1^), tetracycline (TcR, 10 µg mL^−1^), and spectinomycin (Spt, 50 µg mL^−1^).

### 2.2 Plasmids Construction

To construct pCDFDuet‐CDS‐Nudix1, we followed a stepwise process. Initially, the CDS gene was amplified from pET28a‐CDS via PCR using KODone DNA polymerase (TOYOBO, Japan) with gene‐specific primers. It was then inserted into pCDFDuet‐1 between the BamHI/HindIII sites, employing the ClonExpress Ultra One Step Cloning Kit V3 C117‐02 (Vazyme, China), resulting in pCDFDuet‐CDS.

Subsequently, the *Nudix1* gene was amplified from pET28a‐Nudix1 and introduced into pCDFDuet‐CDS between the BglII/XhoI sites to generate pCDFDuet‐CDS‐Nudix1. Similar methodologies were followed to obtain pRSFDuet‐ADH2, pRSFDuet‐ADH2‐ALDH1, pETDuet‐ADH2‐ALDH1, and pRSFDuet‐ADH2‐ALDH1‐ALDH1, pETDuet‐ADH2‐ADH2‐ALDH1, pETDuet‐ispAi, pETDuet‐ADH2‐ALDH1‐ispAi. Plasmid construction was verified by colony PCR (Ultra‐Rapid II HotStart PCR Master Mix 10167ES03, Yeasen) and subsequent Sanger sequencing to confirm the correct gene sequence.

### 2.3 Cultivation of E. Coli Strains

*E. coli* strains (as detailed in Table S2, Supporting Information) were created through the co‐expression of module I plasmids and module II + III plasmids. For shake flask fermentations, the seeds were precultured at 37 °C in LB medium with the appropriate antibiotics overnight. Subsequently, they were inoculated at a 1:100 ratio into 100 mL of LB medium with the necessary supplements. Once the OD_600_ reached the range of 0.6–0.8, IPTG (0.5 mm) was introduced into the cultures. The cultures were then incubated at 16 °C, agitated at 200 rpms, and maintained for 16 h.

Fermenter incubation commenced with the inoculation of the strain into a 1 L fermentation medium, dissolved oxygen (DO) was strictly controlled at or below 30%, serving as the primary control parameter. To improve cell growth and biomass, the concentration of all antibiotics was reduced 50%. Allowing it to proliferate for ≈24 h, achieving an OD_600_ of roughly 8.6. Additionally, a fed‐batch strategy was employed, with a continuous glucose feed to maintain its concentration between 3 and 5 g L^−1^. Following this, gene expression was induced by the addition of IPTG to a final concentration of 1.0 mm. The temperature was held at 20 °C, and a ventilation rate of 2 L min^−1^ was sustained.

### 2.4 Compounds Extraction and Analyses

Culture samples were collected in 10 mL aliquots and extracted with an equivalent volume of methyl tert‐butyl ether (MTBE). After vortex mixing, the mixture was centrifuged at 4000 rpm for 15 min. The supernatant was then concentrated to 1 mL using a nitrogen blower and filtered through a 0.22 filter membrane. To collect the target compounds, MTBE is used to extract the bacterial culture from the fermentation tank, while tetradecane covers the vent hole to facilitate collection. Following MTBE extraction, the sample undergoes vacuum low‐temperature vortex concentration until uniform. The concentrated extract is then redissolved in 1 mL of MTBE for subsequent sample preparation. The standard curve was established by dissolving the standard in the empty vector control culture. This ensured that both the standard curve and all samples were processed using the identical procedure, thereby compensating for both potential matrix effects and any loss of the target compound during extraction and concentration.

Quantification of chrysanthemol, chrysanthemic acid, and other molecules was carried out by gas chromatography‐ mass spectrometry (GC‐MS) analysis using a Thermo Scientific‐TSQ 9000 spectrometer (Thermo Fisher Scientific, U.S.A.) equipped with a DB‐5UI glass capillary column (0.25 mm i.d. × 30 m, 0.25 µm film thickness) from Agilent Technologies in spitless mode. All compounds were quantified by determining their total ion count peak area, followed by calculation using standard curves.

The volatile and semi‐volatile compounds were separated using a gas chromatograph with the following parameters: a temperature program starting at 50 °C, ramping to 280 °C at 10 °C  min^−1^, and then to 290 °C at 4 °C  min^−1^. The injector temperature was set to 270 °C, and a 1 µL sample was injected with a split ratio of 50:1 or in splitless mode. High‐purity helium (99.999%) served as the carrier gas at a flow rate of 1.2 mL min^−1^. The mass spectrometer was operated in full scan mode (m/z 50–500), with the transfer line and ion source temperatures set to 250 and 300 °C, respectively.

Volatile and semi‐volatile compounds were analyzed by GC‐MS. Compounds were identified by matching their mass spectra against the NIST17 database using GC‐rich software. Identification criteria included a response intensity greater than 10^5^, a matching primary ion, and a software confidence level above 90%. The identities of key compounds, such as geraniol and farnesol, were manually verified by comparing their mass spectra to the database.

### 2.5 RNA‐seq and qPCR

For transcriptome analysis, different treated *E. coli* strains were cultured at 20 °C for 72 h. Subsequently, 10 mL of each culture at various growth stages was harvested and frozen in liquid nitrogen for 10 min. All samples were prepared in triplicate. Transcriptome sequencing was performed at BGI (Shenzhen, China). After removing the adapters and low‐quality bases of the sequencing data using SOAPnuke, HISAT2 was used to build the index of the reference genome and align high‐quality RNA‐seq reads. Reads were counted using StringTie, and the gene expression abundance was quantified by Fragments per kilobase of exon per million fragments map (FPKM). Differentially expressed genes based on the number of reads in the samples were identified by the R package DESeq2, and enrichment analysis was played by the R package clusterProfiler. Specific RNA‐seq results are shown in Table S3 (Supporting Information).

Quantitative analysis of target genes was performed using a two‐step process. Following RNA extraction, the integrity and quality of the RNA were assessed by agarose gel electrophoresis. RNA samples were then reverse transcribed into complementary DNA (cDNA) using the AE341 kit. Relative quantification of gene expression was subsequently carried out via qPCR using the AQ602 kit (TransGen Biotech).

### 2.6 Metabolic Modeling

We utilized the preexisting, meticulously curated *Escherichia coli* BL21(DE3) model, iEC1356_Bl21DE3,^[^ ^15^ ^]^ the model was further modified with heterogeneous pathways. Bidirectional BLASTp analysis was conducted between the genomes utilized in the template model (NC_01 2971.2, CP001509.3) and the reference genome (NZ_CP081489.1) employed in our transcriptome sequencing, employing an E‐value threshold of < 1e‐5. Gene IDs of highly matching sequences from NZ_CP081489.1 were mapped onto the template model. For genes not identified through BLAST, we utilized the KEGG and MetaCyc databases, along with relevant literature, to ascertain their gene function and biological information, subsequently integrating them into the model. This iterative process facilitated a preliminary reconstruction of the genome‐scale metabolic network of *Escherichia coli* BL21(DE3).

### 2.7 Model Expansion

In order to enhance the precision and comprehensiveness of the model, we further expanded it by integrating knowledge from various databases (KEGG, MetaCyc, BiGG Models) and metabolic network information gleaned from the literature. First, based on the literature,^[^ ^10^ ^]^ we augmented the BL21_WT model with biochemical reactions from the mevalonate pathway (MVA pathway). This pathway synthesizes isopentenyl diphosphate (IPP) from pyruvate, with IPP serving as a crucial precursor for the synthesis of pyrethroids. Subsequently, following the literature,^[^ ^18^ ^]^ we incorporated downstream biochemical reactions for the synthesis of pyrethroids in the model. These reactions enable the conversion of IPP to chrysanthemic acid, a principal component of pyrethroids. Lastly, leveraging information from literature and databases, we included all possible bypass reactions along the pyrethroid synthesis pathway to account for metabolic competition and the production of by‐products.

Carbon source and biomass composition settings: Since the template model iEC1356_Bl21DE3 and the new model BL21_CS5‐1 simulate the same bacterial strain, the biomass composition in BL21_CS5‐1 remains consistent with the template model. The carbon source settings for BL21_CS5‐1 and BL21_WT were referenced from literature^[^ ^19^ ^]^ and adjusted according to the experimental conditions being simulated.

Model fitting: Absolute quantification of metabolites and expression levels of key pathway genes from different bacterial strains was utilized to further constrain the flux bounds of relevant reactions in the model and the ratios between various fluxes. The COBRA Toolbox^[^ ^20^ ^]^ was employed for model reconstruction and constraint‐based model analysis, utilizing MATLAB R2021a as the computational platform.

### 2.8 Model Analysis and Simulation Details

Flux balance analysis (FBA) was used to compute the maximum growth rate and yield of target products under steady‐state conditions, shown in Equation (1).

(1) $$\max\;V_{\mathrm{biomass}}\;\text{s.t.}\;S\times v=0,\;\mathrm{lb}\leq v\leq \mathrm{ub}$$

where: *V_biomass_* represents the maximum growth rate.*S* represents the stoichiometric matrix of reactions and metabolites in the strain model. *V* represents the reaction flux vector in the strain model. *lb* and *ub* represents the reactions flux bound.

The UniKP^[^ ^21^ ^]^ tool was employed to predict the kinetic parameters of key enzymes in the model (including enzyme turnover number (K_cat_) and Michaelis constant (K_m_)) to calculate the flux distribution centered around candidate gene substrates.

According to the Michaelis–Menten equation, the theoretical maximum reaction rate (V_max_) of any enzymatic reaction is defined as the product of the enzyme's turnover number (K_cat_) and the total enzyme concentration in the cell (E_total_). Consequently, the reaction flux in the model must remain below this maximum value (V_max_) as stipulated by subsequent model constraints. The calculation of the theoretical maximum reaction rate is shown in Equation ((2), (3)).

(2) $$V_{\max}=k_{\mathrm{cat}}\cdot E_{\mathrm{total}}$$

(3) $$E_{\mathrm{total}}=\theta \cdot \mathrm{TPM},\;\text{where}\;\theta >0.$$

where: $V_{\max}$ represents the theoretical maximum reaction rate. $E_{\mathrm{total}}$ represents the total amount of enzymes in the cell. θ represents the optimal weighting factor. *TPM* represents the transcriptional abundance of the corresponding enzyme. We assume the active enzyme concentration is linear to the enzyme transcriptional abundance.

The calculation method for determining the proportion of the catalytic rates (also refer as diverting flux in this paper) of different enzymes acting on the same substrate (DMAPP) is presented in Equation (4).

(4) $$P_{\mathrm{Rate}}=\frac{\frac{k_{\mathrm{cat}}^{j}}{k_{m}^{j}+[s]}}{\sum_{i=1}^{n}\frac{k_{\mathrm{cat}}^{i}}{k_{m}^{i}+[s]}}$$

where: $\;P_{\mathrm{Rate}}$ represents the proportion of the catalytic rates of different enzymes. $k_{cat}^{i}$ enzyme's turnover number for each enzyme. $k_{m}^{i}$ represents Michaelis constant for each enzyme, for means the number of enzymes.

#### 2.8.1 Metabolic Network Modeling to Simulate Gene Knockdown

To simulate the physiological state of the strain under specific culture conditions, the metabolic model BL21_CS5‐1 was subjected to several constraints. First, the upper bound of the glucose uptake flux (reaction *EX_glc__D_e*) was set to 20 mmol/gDW/hr. Second, based on experimental data, the biomass synthesis rate (reaction *BIOMASS_Ec_iJO1366_WT_53p95M*) and the baseline chrysanthemol secretion rate (reaction *EX_CS_OH*) were precisely constrained to 1.706 mmol/gDW/hr and 0.0023 mmol/gDW/hr, respectively. To more accurately reflect the balance of intracellular endogenous metabolic pathways, a proportional constraint was added based on transcriptomic data analysis, fixing the ratio of the mevalonate (MVA) pathway flux (represented by the DPMVD reaction) to the methylerythritol phosphate (MEP) pathway flux (represented by the CDPMEK reaction) at 6461:1.

Additionally, a constraint was implemented to ensure that the total flux of reactions consuming the intermediate dimethylallyl pyrophosphate (DMAPP) did not exceed its initial production rate. As described in Equation (5), where Δ represents the correction factor, which was set to be 0.4:

(5) $$\sum_{i=1}^{m}F_{\mathrm{out}}^{i}\leq \Delta \sum_{j=1}^{n}F_{\mathrm{in}}^{j}$$

whereΔ∈(0,1]

#### 2.8.2 Baseline Flux Calculation and in silico Simulation of *ispA* Gene Knockdown

Initially, Flux Balance Analysis (FBA) was performed on the constrained model with the objective of maximizing biomass synthesis to determine the baseline flux distribution of the CS5 strain. The calculated synthesis fluxes of key intermediates, particularly those catalyzed by terpene synthase (encoded by the *ispA* gene), specifically reactions *DMATT* and *GRTT*, were recorded as baseline values.

Subsequently, to simulate the downregulation of *ispA* gene expression, the fluxes of the reactions catalyzed by *ispA* (DMATT and GRTT) were systematically inhibited in silico. This involved an iterative process where the upper and lower bounds of the DMATT and GRTT reaction fluxes were progressively decreased from their baseline values, simulating inhibition strengths ranging from 2‐fold to 256‐fold.

At each inhibition level, the model's objective function was set as a multi‐objective function, simultaneously maximizing both biomass synthesis and chrysanthemol secretion (reaction *EX_CS_OH*). By solving this optimization problem, the optimal flux state of the metabolic network under different *ispA* activity levels was predicted.

After each iterative simulation, the flux distribution vector of the entire network was recorded, and the predicted chrysanthemol secretion flux (flux_CS) was extracted. To assess the relative impact of *ispA* gene inhibition on target product synthesis, the predicted chrysanthemol flux at each inhibition level was compared to the baseline flux (without inhibition), and the yield improvement ratio was calculated and visualized.

#### 2.8.3 Metabolic Network Modeling to Simulate Gene Overexpression

Using the BL21_CS5‐1 model as a foundation, we performed a reconstruction to simulate and predict the influence of target gene overexpression on target product yield. The workflow began with the determination of the maximum reaction rates for two critical enzymes, ADH2 (represented by E1) and ALDH1 (represented by E2), by integrating their literature‐reported kinetic parameters (turnover number (Kcat​) and Michaelis constant (Km​)) with experimentally measured gene expression levels.

The maximum reaction rate for each enzyme was calculated using the Equation ((3), (6)), where $E_{total}^{i}$ represents the enzyme concentration. The relative in vivo concentrations of enzymes were assumed to be proportional to their measured gene expression levels. This assumption was used to establish an initial constraint on the ratio ($R_{V_{\max}}$) of their maximal reaction rates within the model:

(6) $$R_{V_{\max}}=\frac{V_{\max}^{E1}}{V_{\max}^{E2}}=\frac{K_{\mathrm{cat}}^{E1}\cdot E_{\mathrm{total}}^{E1}}{K_{\mathrm{cat}}^{E2}\cdot E_{\mathrm{total}}^{E2}}=\frac{K_{\mathrm{cat}}^{E1}\cdot \mathrm{EX}P_{E1}}{K_{\mathrm{cat}}^{E2}\cdot \mathrm{EX}P_{E2}}$$

This constraint effectively links the model's flux capacities to biological measurements.

Following the initial model setup, this ratio constraint was removed to explore a broader theoretical landscape of enzyme expression. The model was re‐simulated under a range of conditions where the upper bounds of the fluxes for reactions *f1* and *f2* were systematically varied. These upper bounds were defined as *nf1* and *nf2*, where *n* represents a scaling factor. By systematically altering the value of *n*, we simulated the impact of varying enzyme expression levels on the network's capacity for product synthesis.

### 2.9 Statistics and Reproducibility

All experiments were conducted with *N* ≥ 3 independent biological replicates. Sample sizes (*n*) for other experiments and analyses are specified in the corresponding figure legends. Transcriptomic and metabolomic data were normalized using z‐score normalization and analyzed with the R statistical package. Metabolite quantification and statistical comparisons were performed using two‐tailed *t*‐tests, with all *P*‐values being <0.05.

## 3 Results

### 3.1 Establishing a Robust Chrysanthemic Acid Biosynthesis Chassis Through Modular Engineering and Modelling‐Guided Optimization

Three modules were designed to establish a robust chassis for chrysanthemic acid biosynthesis (Figure 1B). First, to ensure a continuous supply of DMAPP, the crucial precursor of chrysanthemic acid, the exogenous MVA pathway was introduced via co‐expression of pre‐validated pMevT and pMBI plasmids in *E. coli* BL21(DE3) (CS3). Subsequently, CS5‐1 strain was generated by introducing the second module responsible for chrysanthemol biosynthesis, which includes scaffold‐forming gene CDS and Nudix1 from pyrethrum. For the final production of chrysanthemic acid, a third module was incorporated into CS5‐1 through the introduction of two dehydrogenases from pyrethrum, ADH2 and ALDH1, leading to strain CS6.

To further augment chrysanthemic acid yield, a comprehensive series of strategic optimizations was executed, critically informed by metabolic modeling of the engineered strains. This modeling integrated multi‐omics data, enabling the precise identification of key metabolic flux nodes and facilitating the rational redirection of metabolic flow. Specifically, these optimizations encompassed synthetic sRNA‐mediated repression of the ispA gene (strains CS5‐3 and CS11) and fine‐tuning the copy numbers of *ADH* and *ALDH* genes (strains CS7‐CS10). Detailed elaboration of these optimization strategies will be provided in subsequent sections (Figure 1B).

### 3.2 Genome‐Scale Metabolic Modeling Integrated with Multi‐Omics for Analyzing Metabolic Perturbations by a Heterologous MVA Pathway

**Figure 2:**
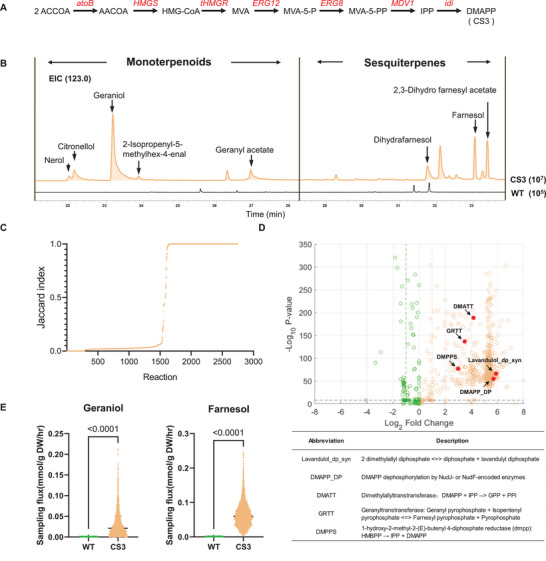
Flux distribution analysis using GEM. A) The biosynthetic pathway of DMAPP originates from Acetyl‐CoA. B) Types of terpenes produced by the CS3 strain (EIC = 123.0). C) Sorting results of FVA Jaccard index between BL21_WT and BL21_CS3. D) Volcano plot of reaction fluxes from flux sampling in the WT and CS3 models. The x‐axis (log_2_FC) shows the fold change between the two groups, with positive values indicating upregulation and negative values indicating downregulation. The y‐axis (‐log_10_ p‐value) reflects statistical significance, with higher values indicating stronger significance. A significance threshold is set at p‐value < 10^−^⁸, with the black dashed line at ‐log_10_ p‐value = 8. The fold change reference lines are green at log_2_FC = ‐1 and yellow at log_2_FC = 1, corresponding to absolute fold changes greater than 2 (|log_2_FC| > 1). E) Comparison of flux‐sum values for reactions producing geraniol and farnesol under flux sampling in both the WT and CS3 models. DMAPP: dimethylallyl diphosphate; DMAP: dimethylallyl monophosphate; GPP: geranyl diphosphate; FPP: farnesyl diphosphate.

The MVA pathway was reconstructed by co‐expression of 7 related genes from *S. cerevisiae* and *E. coli* (**Figure** **2**A). Analyzed via GC‐MS, the engineered CS3 strain exhibited high concentrations of various monoterpenes and sesquiterpenes, including geraniol and farnesol, compared to the wild‐type strain (Figure 2B and Figure S1, Supporting Information). While this result confirmed an abundant supply of terpene precursors, the substantial accumulation of these volatile terpenes highlighted the critical need for the optimization of the metabolic network in this chassis.

To simulate metabolic flux distribution under the metabolic perturbations induced by the heterologous MVA pathway, transcriptomic and metabolomic data were collected and analyzed. A comprehensive dataset comprising 45 samples from five strains (wild‐type, CS3, CS4, CS5‐1, and CS6) was collected at three timepoints (before induction, 16 h post‐induction, and 48 h post‐induction), with three biological replicates per strain, for RNA sequencing (RNA‐Seq). Gene expression profiles across the different strains were acquired (Figure S2, Supporting Information). Principal Component Analysis (PCA) of these profiles revealed a clear separation between the wild‐type and experimental strains at the mid‐induction stage (Figure S3, Supporting Information). Furthermore, differential gene expression patterns (Figure S4, Supporting Information), alongside Gene Ontology (GO) and KEGG enrichment analyses (Figures S5–S8, Supporting Information), indicated a significant upregulation in carbon and secondary metabolism in the experimental strains compared to the wild‐type.

In parallel, the volatile metabolome of the corresponding strains was measured via GC‐MS. As anticipated, the majority of metabolites in the experimental strains resembled those in the control group, with alterations primarily confined to a subset of terpenoid metabolic pathways, exhibiting minimal impact on primary metabolism (Figure S9, Supporting Information). This observation was further supported by PCA of the metabolome data (Figure S10, Supporting Information). Differential metabolite analysis revealed that the introduction of an exogenous terpenoid metabolic pathway significantly impacted the biosynthesis of a limited number of compounds in the experimental strains compared to the control group (Figures S11–S13, Supporting Information). Concurrently, Orthogonal Partial Least Squares Discriminant Analysis (OPLS‐DA) demonstrated that compounds synthesized via the introduced terpene pathway, such as geraniol and farnesol, significantly influenced the separation of strains in the OPLS‐DA plot (Figure S14 and Table S4, Supporting Information).

The acquired omics data were subsequently utilized for the construction of a metabolic model. Detailed information regarding the metabolites and reactions added to the original iEC1356_Bl21DE3 model is provided in the supplementary material. The quality of models BL21_WT, BL21_CS3, and BL21_CS5‐1 was rigorously evaluated using MEMOTE, a standardized Genome‐scale Metabolic Model (GEM) quality check tool.^[^ ^22^ ^]^ The generated reports confirmed the stoichiometric consistency and mass balance of these models (MEMOTE_REPORT). Subsequently, Flux Variability Analysis (FVA)^[^ ^23^ ^]^ and Flux Sampling^[^ ^24^ ^]^ were employed to investigate differences in metabolic pathway distribution between the wild‐type (WT) strain and the CS3 strain, which incorporated the MVA pathway (Figure 2C,D).

The introduction of the MVA pathway not only influenced the terpene biosynthesis pathway but also affected more than half of *E. coli*’s metabolic network. Subsequently, Flux Sampling results predicted that the MVA pathway in the CS3 strain could branch flux from the chrysanthemol synthetic pathway toward by‐product (C10, C15 terpenes) production, including geraniol and farnesol (Figure 2E). This prediction was consistent with the observed increases in monoterpenoids and sesquiterpenes detected by ion chromatography (Figure 2B). These modeling results collectively imply that debranching flux from competitive pathways can redirect by‐product biosynthesis toward chrysanthemol production, thereby leading to an increase in chrysanthemol yield.

### 3.3 Model‐Driven Optimization of Metabolic Network and Redirection of DMAPP Flux Toward Chrysanthemol Biosynthesis

**Figure 3:**
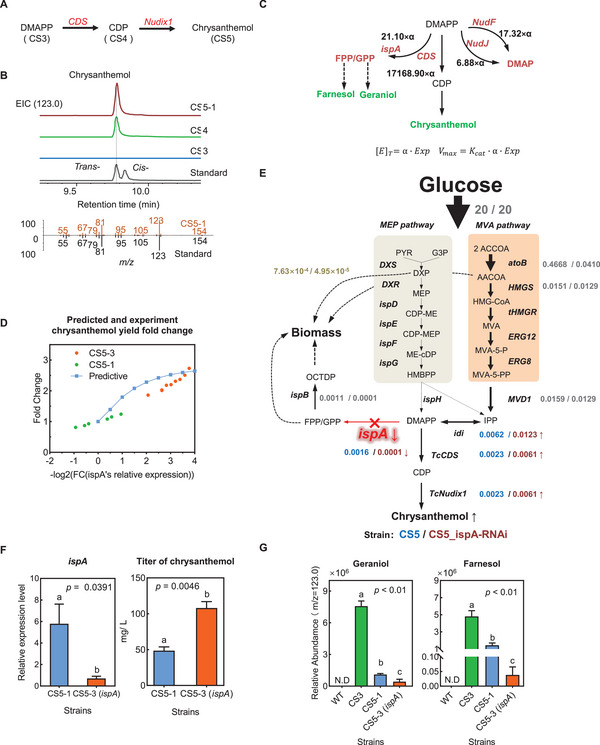
Synergistic Metabolic Modeling and Experimental Approaches Guide Optimized Chrysanthemol Biosynthesis. A) The chrysanthemol biosynthesis pathway originates from DMAPP. B) Introduction of MVA and *CDS* gene strain samples and chrysanthemol standard (EIC = 123.0) and mass spectrometry information of chrysanthemol (Orange: CS4 strain, Black: Standard). C) Schematic representation of the biosynthetic network for the target product chrysanthemol and its competing byproducts. The pathways for all byproducts, including geraniol, farnesol, and DMAP, compete for the key precursor DMAPP. The numerical values on each pathway represent the absolute maximum reaction rate (Vmax). These Vmax values were calculated from the turnover number (Kcat) of the respective enzymes, providing a direct comparison of the kinetic capacities of the main and byproduct‐forming pathways. D) The effects of simulated and experimental inhibition of *ispA* expression in CS5‐1 strain on chrysanthemol titer. E) Simulated flux distribution in Strain CS5‐1 (left) and Strain CS5‐3 (right) models using FBA (Flux Balance Analysis). Simulated flux values for the corresponding strains are represented by blue and yellow numbers, respectively. F) qPCR of *ispA* gene and chrysanthemol titer of CS5‐3 strain constructed by gene silencing of *ispA* gene (N = 3). G) Relative abundance of geraniol and farnesol in different strains (EIC = 123.0, N = 3). G3P, Glyceraldehyde 3‐phosphate; Pyr, Pyruvate; DXP, 1‐Deoxy‐D‐xylulose 5‐phosphate; MEP, 2‐C‐Methyl‐D‐erythritol 4‐phosphate; CDP‐ME, 4‐Diphosphocytidyl‐2‐C‐methyl‐D‐erythritol; CDP‐MEP, 4‐diphosphocytidyl‐2‐C‐methyl‐D‐erythritol; ME‐CDP, 2‐C‐Methyl‐D‐erythritol 2,4‐cyclodiphosphate; HMBPP, (E)‐4‐Hydroxy‐3‐methyl‐but‐2‐enyl pyrophosphate; DMAPP, Dimethylallyl pyrophosphate; IPP, Isopentenyl‐5‐pyrophosphate; GRDP, Geranyl diphosphate; FRDP, Farnesyl diphosphate; OCTDP, All‐trans‐octaprenyl diphosphate; AACOA, Acetoacetyl‐CoA; ACCOA, Acetyl‐CoA; HMG‐CoA, (3S)‐Hydroxy‐3‐methylglutaryl‐CoA; MVA, (R)‐Mevalonate; MVA‐5‐P, Mevalonate‐5‐phosphate; MVA‐5‐PP, Mevalonate‐5‐pyrophosphate; CDP, (R,R)‐Chrysanthemyl diphosphate.

Chrysanthemol biosynthesis relies on DMAPP as a substrate, with CDS and Nudix1 catalyzing the conversion (**Figure** **3**A). Expressing CDS alone in the CS3 (strain CS4) leads to the production of 33.31 mg L^−1^ chrysanthemol after 48‐h incubation (Figure 3B). The extracted ion chromatogram (EIC) for chrysanthemol was generated at m/z 123.0. Although the molecular ion (m/z 154.2) is present, the fragment ion at m/z 123.0 exhibits a significantly higher response intensity, which provides enhanced sensitivity and enables more robust detection of the compound (Figure 3B). Hydrolyzation of chrysanthemyl diphosphate to chrysanthemol in this strain may be due to the promiscuity of phosphohydrolases from wild‐type *E. coli*. To enhance chrysanthemol production, we further introduced pyrethrum Nudix1 phosphohydrolase into the CS4 strain (strain CS5‐1). The fermentation of the CS5‐1 yielded 44.69 mg L^−1^ chrysanthemol within 48 h, 134.16% of the titer obtained from the CS4 strain (Figure S15, Supporting Information).

To identify the critical reaction responsible for diverting DMAPP flux away from chrysanthemol production, the flux distribution at the metabolic crossroads of DMAPP was simulated. Initially, the maximum reaction rates (Vmax) of various branching reactions involving the precursor metabolite DMAPP within its synthesis pathway were calculated (Method 2.8). This was achieved by leveraging transcriptional data and employing the machine learning‐based turnover rate prediction algorithm, UniKP^[^ ^21^ ^]^ tool. The results indicated that the Vmax of ispA‐catalyzed DMAPP utilization was the highest among the identified branching reactions (Figure 3C). Subsequently, the flux ratios of different enzymes interacting with the substrate DMAPP were computed across varying orders of substrate concentration. Computational analysis revealed that, across diverse substrate concentration levels, the diverting flux ratio of ispA to DMAPP was notably higher than that of other enzymes involved in alternative bypass biosynthetic pathways (Figure S16 and Table S5, Supporting Information). The hypothesis posits that farnesyl diphosphate synthase (ispA), which catalyzes the synthesis of GPP and FPP from DMAPP and IPP in *E. coli*, significantly diverts the key precursor metabolite DMAPP in strain CS5‐1.

Consequently, a debranching strategy involving the suppression of ispA expression was proposed to enhance chrysanthemol titer. However, consistent with previous literature reports, our model simulations of ispA knockout yielded no biomass production, which indicates ispA is essential for *E. coli* growth and that its direct knockout results in strain lethality.^[^ ^25^ ^]^ ispA gene inhibition was simulated in the BL21_CS5‐1 model using Flux Balance Analysis (Figure 3D,E). Prior to simulation, specific constraints were applied to the CS5‐1 model: glucose was designated as the sole carbon source with an uptake rate limit of 20 mmol/g·DW/h. Furthermore, because ispA is critical for cell growth, the total flux of the MEP and MVA pathways, as well as GPP and FPP flux, was initially constrained based on experimental data related to cell growth and chrysanthemol yield.

To simulate possible effects of ispA knockdown on cell growth and chrysanthemol production, we partially constrained ispA flux (as detailed in formula (4) and Figure S17, Supporting Information). The predicted results indicated a 2.64‐fold increase in chrysanthemol total flux under conditions where ispA expression was reduced to 1/16th of that in CS5‐1, while maintaining cell viability (Figure 3D,E, and Figure S18, Supporting Information). This model simulation thus indicated that ispA expression inhibition can remarkably enhance chrysanthemol production and circumvent the lethal phenotype, thereby underscoring the efficacy of a “debranching” strategy for redirecting flux toward target products.

To experimentally confirm the model's predictions, ispA expression was inhibited in strain CS5‐1, generating strain CS5‐3, through the application of a synthetic small RNA within a heterologous RNA interference system in *E. coli*. Quantitative PCR (qPCR) verified the success of this inhibition, revealing ispA expression levels in CS5‐3 that were 87.77% lower than those in the CS5‐1 control (Figure 3F). Concurrently, the chrysanthemol content in both strains was quantified. After 48 h of cultivation, CS5‐3 exhibited a significantly higher chrysanthemol concentration (117.03 mg L^−1^), representing an ≈119.83% increase over strain CS5‐1′s 53.24 mg L^−1^ (Figure 3F). This result aligns perfectly with GEM's prediction, demonstrating that the chrysanthemol titer increased as ispA expression decreased. Furthermore, strain CS5‐3 exhibited markedly lower concentrations of both geraniol (60.94% reduction) and farnesol (97.32% reduction) compared to strain CS5‐1 (Figure 3G). These findings conclusively validate that inhibiting ispA expression is a feasible and critical strategy for significantly boosting chrysanthemol production.

### 3.4 Fermentation Optimization for High Chrysanthemol Production

To efficiently explore multiple optimization strategies and reduce overall experimental time, we conducted a series of trials in strain CS5‐1 to optimize chrysanthemol production conditions—including incubation time, temperature, inducer concentration, and culture medium—in parallel with the optimization of metabolic flux. An intermittent sampling strain was conducted for 102 h to supervise its growth and production curves. The result indicated that the density of cells reached the peak at 26 h, but the chrysanthemol still continuously accumulated in the culture (Figure S19A, Supporting Information). Further trial indicated that the best IPTG concentration and temperature were 1.0 mm and 20 °C in 2 ×YT media (Figure S19B–D, Supporting Information). Combined with the above optimization conditions, the CS5‐1 strain achieved a remarkable yield of 80.6 mg L^−1^ of chrysanthemol under specific conditions after 54‐h fermentation (Figure S19G, Supporting Information). Further analysis showed the distribution of chrysanthemol between intracellular and extracellular, and ≈95% was present in the medium (Figure S19E, Supporting Information). This result may be attributed to the free diffusion of chrysanthemol across the cell membrane, facilitated by its lipophilic and low molecular weight.

Building upon the CS5‐1, we attempted to optimize the cDNA sequence of *CDS* and *Nudix1* by *E. coli* codons (strain CS5‐2). Unfortunately, the optimized CS5‐2 strains obtained even lower level of chrysanthemol (Figure S19F, Supporting Information). This outcome aligned with previous studies that altering codon usage patterns might not always lead to increased target product yield and emphasized the complexities of gene optimization.^[^ ^26^ ^]^ The overexpression of optimized genes could result in the formation of inclusion bodies, which are often associated with misfolded or aggregated proteins.

### 3.5 Model‐Guided Optimization of ADH2 and ALDH1 Copy Numbers for Enhanced Chrysanthemic Acid Production

**Figure 4:**
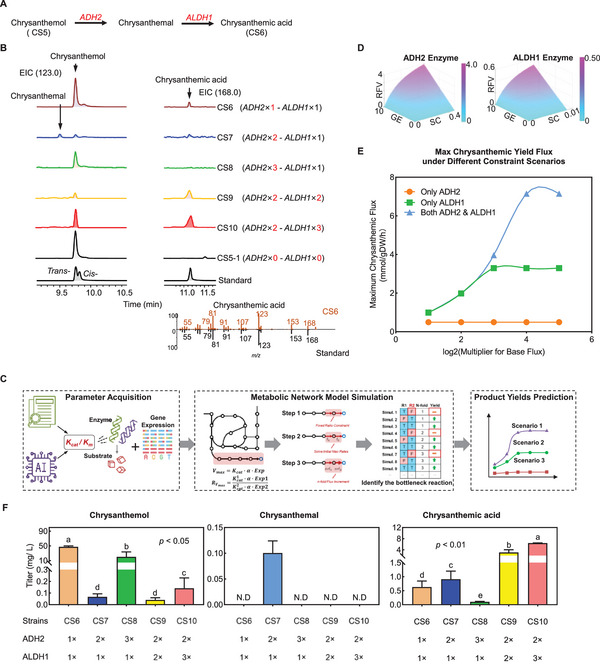
Construction and optimization of the chrysanthemic acid module. A) Chrysanthemic acid biosynthesis pathway, starting from chrysanthemol. B) Top: EIC of chrysanthemol, chrysanthemal, and chrysanthemic acid in strains with different *ADH2* and *ALDH1* copy numbers (N = 3). Botten: mass spectrometry information of chrysanthemic acid (black: standard; orange: CS6 strain). C) Metabolic network modeling workflow diagram. D) Predicted enzyme reaction flux as a function of substrate concentration and gene expression x: substrate concentration [S] (mM); y: gene expression; z: reaction flux V (mM·s^−1^​). E) Simulated optimization of chrysanthemic acid production via enzyme flux manipulation. F) Titer of chrysanthemol, chrysanthemal, and chrysanthemic acid with different *ADH2* and *ALDH1*copy numbers (N = 3).

Subsequent efforts concentrated on the chrysanthemic acid biosynthesis module (Module III), which encompasses two oxidative steps from chrysanthemol (**Figure** **4**A). Initial engineering of strain CS5‐1 with dehydrogenases ADH2 and ALDH1 (via pRSFDuet‐ADH2‐ALDH) enabled the detection of 0.63 mg L^−1^ chrysanthemic acid from chrysanthemol in strain CS6 (Figure 4B,F). However, a substantial 49.30 mg L^−1^ of chrysanthemol remained unconverted, highlighting the necessity for optimizing ADH2 and ALDH1 expression for enhanced substrate conversion and yield (Figure 4F).

To identify the bottleneck reaction of chrysanthemic acid biosynthesis, the GEM of CS6 and the Michaelis–Menten Equation were employed to compare ADH2 and ALDH1 dynamic reaction rates. A metabolic network modeling workflow diagram was first established (Figure 4C). First, initial maximum reaction rates for two enzymes were calculated by integrating their literature‐reported enzyme kinetics (Kcat and Km) with experimentally determined gene expression levels, which served to constrain their maximal reaction rate ratio (Rv_max_) within the metabolic network model^[^ ^8^ ^]^ (Equation (6)). Second, to identify the bottleneck reaction, reaction expression enhancement strategies, such as n‐fold gene expression increase with the combination of genes, were simulated to predict product yield. The maximum reaction rate of ALDH1 was less than ADH2 (Figure 4D). These models then simulated the theoretical maximal flux under various overexpression strategies, providing critical insights: increasing ALDH1 flux alone robustly elevated chrysanthemic acid yield to ≈3.3 mmol/g·DW/h (green curve), whereas increasing ADH2 flux alone exerted no discernible impact (orange curve). Crucially, the model predicted that simultaneous optimization of both ALDH1 and ADH2 resulted in a synergistic amplification, pushing the yield to ≈7 mmol/g·DW/h (blue curve) (Figure 4E). This underscored the imperative for co‐regulating both enzymes to overcome identified bottlenecks and substantially enhance chrysanthemic acid production.

Following computational prediction, the impact of *ADH2* gene copy numbers on chrysanthemic acid production was experimentally assessed. Strains engineered with one, two, or three ADH2 copies were characterized. The double‐copy strain (CS7) showed ≈5.4‐fold higher ADH2 expression than the single‐copy strain (Figure S20A, Supporting Information). While this led to a significant reduction in chrysanthemol, indicating its increased consumption, overall chrysanthemic acid production did not greatly increase. Concomitantly, chrysanthemal was detected, highlighting a persistent bottleneck in the subsequent conversion step (Figure 4B,F). The decrease in chrysanthemal titer at higher ADH2 copy numbers may stem from lower ADH2 expression, as seen in strain CS8 compared to CS7 (Figure S20A, Supporting Information). We speculate this is caused by transcriptional‐translational coupling, where an excess of gene copies leads to a “traffic jam” on the mRNA, thus reducing overall protein synthesis. This empirical observation aligns with the computational model's prediction that increasing ADH2 flux in isolation yields a limited impact, thereby emphasizing the necessity for synergistic optimization of both ALDH1 and ADH2.

Based on these combined simulation and preliminary experimental outcomes, ALDH1 was identified as the primary rate‐limiting enzyme in chrysanthemic acid synthesis. To experimentally validate this hypothesis and further enhance chrysanthemol conversion to chrysanthemic acid, ALDH1 copy numbers were subsequently optimized in the CS7 strain backbone. Evaluation of strains with increased ALDH1 copy numbers (CS9 with two, CS10 with three, vs CS7 with one) demonstrated that ALDH1 expression levels were significantly higher in CS10 (120% increase compared to CS7) (Figure S20B, Supporting Information). Our qPCR results show that while the expression of ALDH1 was lower in strain CS9 than in CS7 at the 16‐h time point, the temporal expression profile of ALDH1 differs between the two strains. Our measurements at 4‐, 8‐, 12‐, and 16‐h post‐induction revealed that the peak expression of ALDH1 occurred earlier in CS9 than in CS7. This earlier expression could be a key factor contributing to the higher final chrysanthemic acid yield in CS9, even though its expression level was lower than CS7 at the final time point (Figure S21, Supporting Information). Concurrently, the introduction of three copies of the ALDH1 gene in CS10 dramatically improved chrysanthemol conversion, achieving a chrysanthemic acid yield of 6.48 mg L^−1^ in 16 h, a 5.7‐fold increase compared to the single‐copy ALDH1 strain (Figure 4B,F). This experimental result precisely corroborated the model predictions, unequivocally confirming ALDH1 as the key bottleneck in the pathway.

### 3.6 Synergistic Optimization for Enhanced Chrysanthemic Acid Production

**Figure 5:**
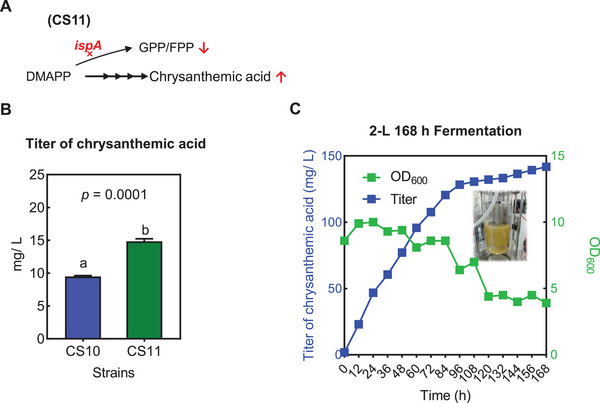
Chrysanthemic acid production by CS11 strain. A) Schematic of strain CS11 construction. B) Chrysanthemic acid titer of strain CS11 in shake flask culture (N = 3). C) Chrysanthemic acid production in 2L fermenter scale‐up.

To further augment chrysanthemic acid yield, an integrated strategy was implemented combining upstream debranching with enhanced downstream conversion. Strain CS11 was constructed by introducing the ispA silencing system (previously optimized in CS5‐1) into the copy number‐optimized CS10 strain (**Figure** **5**A). Under the established optimal culture conditions for chrysanthemic acid production, strain CS11 achieved a yield of 15.30 mg L^−1^ within 48 h, compared to 9.59 mg L^−1^ for CS10 (Figure 5B).

To evaluate scalability, preliminary experiments were subsequently conducted in a 2L bioreactor, employing n‐tetradecane for collection. Dissolved oxygen (DO) was controlled at 30% by cascading with the agitation speed, which was allowed to vary between 400 and 1000 rpm. The pH was maintained at 7.0 by the automatic addition of an ammonia solution. A fed‐batch strategy was employed using a 0.5 g mL^−1^ glucose solution, with the glucose concentration maintained between 3–5 g L^−1^ throughout the feeding phase. This process ultimately resulted in a chrysanthemic acid yield of 141.78 mg L^−1^ at 168 h (Figure 5C), representing the highest production level attained to date.

## 4 Discussion

As modern agriculture's output grows, so does the demand for pesticides.^[^ ^27^ ^]^ With the rise of synthetic biology, there's a growing need for environmental protection and sustainable practices.^[^ ^28^ ^]^ As the extensive use of chemically synthesized pyrethroids, such as fenpropidin and delta permethrin, resistance in insects to those increases rapidly, while natural pyrethrins remain potent.^[^ ^29^, ^30^, ^31^ ^]^

Chrysanthemic acid, a key component of pyrethrins, possesses natural insect‐repelling properties. Although the metabolic pathways of pyrethrins are not completely understood, the development of microbial cell factories for chrysanthemic acid serves as a foundation for pyrethrin production or as a standalone insecticide, offering promising ecological benefits. Our study is dedicated to making use of the elucidated genes involved in pyrethrin synthesis for microbial engineering, aiming to reduce the production costs of pyrethrin. We have successfully synthesized chrysanthemic acid, the acid moiety of pyrethrin, in *E. coli*, and with a higher yield compared with the chrysanthemic acid yield in chrysanthemum^[^ ^3^ ^]^ and in tomato.^[^ ^9^ ^]^

Optimal metabolic engineering requires a systematic understanding of the designed metabolic pathways and regulatory mechanisms. In our work, we completed the production of chrysanthemol in *E. coli* by introducing the chrysanthemol synthesis gene. Through the integration of metabolomics and transcriptomics, we concluded that the diversion of DMAPP limits the production of pyrethrin in this host. To overcome this limitation, we constructed a high‐resolution metabolic model by combining transcriptomics and metabolomics data and predicted the branching pathway of DMAPP. Comparison of the production of chrysanthemol and chrysanthemic acid in *E. coli* with the inhibited expression of *ispA* using synthetic small RNA and *E. coli* with uninhibited *ispA* expression showed that the increase of synthesized chrysanthemol and chrysanthemic acid by 162% and 59% respectively.

Using our GEM model, we predicted the key rate‐limiting step in chrysanthemic acid synthesis. The model uncovered an unexpected phenomenon: co‐enhancing key rate‐limiting and non‐rate‐limiting enzymes significantly raised the yield ceiling compared to upregulating them individually. Experimental validation confirmed this predicted synergistic effect, highlighting the model's potential to guide efficient metabolic pathway engineering. Specifically, increasing the copy number of *ADH* and *ALDH*, combined with metabolic model predictions and gene silencing, resulted in an 23.29‐fold increase in chrysanthemic acid accumulation after 48 h.

Our metabolic models, which integrated genomic, biochemical, and metabolic network data, proved valuable for predicting gene knockout impacts and identifying overexpression targets, demonstrating their utility even with limited data. However, these models have limitations, including their static nature, which omits dynamic regulatory information, neglecting the dynamic nature of transcriptional regulation and changes in enzyme activity (such as allosteric effects), which can lead to biased predictions, particularly for transient metabolic responses. Another limitation is the absence of comprehensive kinetic parameters for certain enzymes that are not fully studied. For future work, we plan to improve model accuracy by integrating multi‐omics data and gene regulation patterns generated by an artificial intelligence model to better constrain the models.^[^ ^33^, ^34^ ^]^ Furthermore, the integration of thermodynamic constraints will be considered to further refine model accuracy.

In conclusion, our study took advantage of model‐guided metabolic engineering approaches and the highly accurate predictions of cellular phenotypes it offered, significantly reducing the experimental costs and time associated with developing productive microbial strains. This methodology assists in designing more rational and efficient strategies for metabolic engineering.

In addition to these techniques, we have also utilized traditional metabolic engineering methods, such as culture medium optimization, temperature control, and induction intensity optimization, to improve chrysanthemic acid production. We achieved a pyrethrin output of 141.78 mg L^−1^, marking the highest reported yield to date.^[^ ^8^, ^9^, ^32^ ^]^ The bacterial culture's optical density (OD) was lower than expected during bioreactor fermentation, with a slow decline in cell density observed after induction. We hypothesize that this could be due to three factors. First, the five plasmids and their corresponding antibiotic resistance markers in strain CS11 imposed a significant metabolic burden, slowing the growth rate. Second, the repression of the *ispA* gene, a strategy used to redirect metabolic flux, is known to inhibit cell growth upon inducer activation. Finally, the accumulation of chrysanthemic acid during the later stages of fermentation may have caused cytotoxicity, also contributing to the decrease in cell density. Solving those problems will improve the titer of chrysanthemic acid in the future. Our work offers valuable references and insights for the biosynthesis of natural products.

## 5 Conclusion

In this study, the *E. coli* chassis for chrysanthemic acid biosynthesis was constructed by introducing catalytic genes from the pyrethrum plant. To increase the production rate, the Genome‐scale metabolic model simulated metabolic perturbation according to the time course transcriptome and metabolome. As predicted by the model, the key bypass pathway was identified and partially blocked, leading to a 162% and 59% increase in products chrysanthemol and chrysanthemic acid, respectively. To enhance downstream conversion efficiency, the model guided the identification of the rate‐limiting step, leading to a 470% yield increase via optimized key gene copy numbers. The titer of chrysanthemic acid reached a recorded 141.78 mg L^−1^ in the bioreactor. Two key factors that limit the product of chrysanthemol and chrysanthemic acid in *E. coli* are the debranching pathway and the downstream conversion rate.

## Conflict of Interest

The authors declare no conflict of interest.

## Supporting information



Supporting Information

Supplemental Table 3

Supplemental Table 4

Supplemental Table 6

Supporting Data

## Data Availability

The data that support the findings of this study are available from the corresponding author upon reasonable request.
