# Supplementary material for: Genome‐scale Metabolic Modeling Guided Escherichia coli Engineering for De Novo Biosynthesis of Chrysanthemic Acid
Source: Adv Sci (Weinh). 2025 Nov 21;13(2):e12736. doi: 10.1002/advs.202512736 (PMC12786348; doi:10.1002/advs.202512736)
Supplement: Supplementary file 1 — Supporting Information [file ADVS-13-e12736-s001.docx]

**SUPPLEMENTARY DATA**

**Genome-scale metabolic modeling guided *Escherichia coli* engineering for *de novo* biosynthesis of chrysanthemic acid
*Jiangpeng Yu^a, b, 1^, Kelin Cheng^a, 1^, Shenyang Qu^a^, Jie Wang^a^, Xun Wang^c^, Cheng Zhao^a, *^, Wei Li^a, *^***

^a^ Shenzhen Branch, Guangdong Laboratory of Lingnan Modern Agriculture, Key Laboratory of Synthetic Biology, Ministry of Agriculture and Rural Affairs, Agricultural Genomics Institute at Shenzhen, Chinese Academy of Agricultural Sciences, Shenzhen 518124, China

^b^ College of plant & technology, Huazhong Agricultural University, Wuhan 430070, People’s Republic of China

^c^ National Key Laboratory of Agricultural Microbiology, College of Life Science and Technology, Huazhong Agricultural University, Wuhan 430070, People’s Republic of China

^1^ These authors contributed equally.

* Corresponding author: [liwei11@caas.cn](mailto:liwei11@caas.cn), [zhaocheng01@caas.cn](file:///C:\SynologyDrive\课题组成员\余江鹏\zhaocheng01@caas.cn)

**Table S1. Plasmids used in this study.**

| **Designation** | **Characteristics** | **Reference** |
| --- | --- | --- |
| pMevT | p15A *ori*, Cm^R^, atoB, tHRMG, HMGS | (Martin, et al., 2003) |
| pMBI | pBBR1 *ori*, Tc^R^, ERG12, ERG8, MVD1, idi | (Martin, et al., 2003) |
| pCDFDuet-1 | CloDF13 *ori,* Sm^R^ | Novagen# 71340 |
| pRSFDuet-1 | RSF *ori*, Kan^R^ | Novagen# 71341 |
| pETDuet-1 | f1 *ori*, Amp^R^ | Novagen# 71146 |
| pCDFDuet-CDS | pCDFDuet-1 derivative containing the CDS gene | This study |
| pCDFDuet-CDS-Nudix1 | pCDFDuet-1 derivative containing the CDS and Nudix1 genes | This study |
| pCDFDuet-opCDS-opNudix1 | pCDFDuet-1 derivative containing the Optimized CDS and Optimized Nudix1 genes | This study |
| pETDuet-ispA | pETDuet-1 derivative containing the silencing module of ispA | This study |
| pRSFDuet-ADH2 | pRSFDuet-1 derivative containing the ADH2 gene | This study |
| pRSFDuet-ADH2-ALDH1 | pRSFDuet-1 derivative containing the ADH2 and ALDH1gene | This study |
| pRSFDuet-ADH2-ADH2-ALDH1 | pRSFDuet-1 derivative containing the ADH2×2 and ALDH1 gene | This study |
| pETDuet-ADH2 | pETDuet-1 derivative containing the ADH2 gene | This study |
| pETDuet-ADH2-ALHD1 | pETDuet-1 derivative containing the ADH2 and ALDH1gene | This study |
| pETDuet-ADH2-ALHD1-ALDH1 | pETDuet-1 derivative containing the ADH2 and ALDH1 ×2 gene | This study |
| pETDuet-ADH2-ispA-ALHD1-ALDH1 | pETDuet-1 derivative containing the ADH2, ALDH1 ×2 and the silencing module of ispA | This study |

**Table S2. Strains used in this study.**

| **Designation** | **Characteristics** | **Reference** |
| --- | --- | --- |
| *E. coli* Top10 | Cloning strain, cultured at 37 °C | WEIDI |
| *E. coli* BL21(DE3) | Working strain | WEIDI |
| CS1 | *E. coli* BL21(DE3) harboring pMevT | This study |
| CS2 | *E. coli* BL21(DE3) harboring pMBI | This study |
| CS3 | *E. coli* BL21(DE3) harboring pMevT and pMBI | This study |
| CS4 | *E. coli* BL21(DE3) harboring pMevT, pMBI and pCDFDuet-CDS | This study |
| CS5-1 | *E. coli* BL21(DE3) harboring pMevT, pMBI and pCDFDuet-CDS-Nudix1 | This study |
| CS5-2 | *E. coli* BL21(DE3) harboring pMevT, pMBI and pCDFDuet-opCDS-opNudix1 | This study |
| CS5-1-ipsA | *E. coli* BL21(DE3) harboring pMevT, pMBI, pCDFDuet-CDS-Nudix1 and pETDuet-ispAR | This study |
| CS6 | *E. coli* BL21(DE3) harboring pMevT, pMBI, pCDFDuet-CDS-Nudix1 and pRSFDuet-ADH2-ALDH1 | This study |
| CS7 | *E. coli* BL21(DE3) harboring pMevT, pMBI, pCDFDuet-CDS-Nudix1 and pRSFDuet-ADH2-ADH2-ALDH1 | This study |
| CS8 | *E. coli* BL21(DE3) harboring pMevT, pMBI, pCDFDuet-CDS-Nudix1, pRSFDuet-ADH2-ADH2-ALDH1 and pETDuet-ADH2 | This study |
| CS9 | *E. coli* BL21(DE3) harboring pMevT, pMBI, pCDFDuet-CDS-Nudix1, pRSFDuet-ADH2-ADH2-ALDH1 and pETDuet-ADH2-ALDH1 | This study |
| CS10 | *E. coli* BL21(DE3) harboring pMevT, pMBI, pCDFDuet-CDS-Nudix1, pRSFDuet-ADH2-ADH2-ALDH1 and pETDuet-ADH2-ALDH1-ALDH1 | This study |
| CS11 | *E. coli* BL21(DE3) harboring pMevT, pMBI, pCDFDuet-CDS-Nudix1, pRSFDuet-ADH2-ADH2-ALDH1 and pETDuet-ADH2-ispAR-ALHD1-ALDH1 | This study |

**Table S5. UniKP predicts results and maximum reaction rate.**

| **Gene ID** | **Description** | **Proteins name** | **Kcat** | **Km** | **TPM** | **Vmax** |
| --- | --- | --- | --- | --- | --- | --- |
| K5T46_RS00410 | DMAPP dephosphorylation | NudF | 2.95248 | 0.03326 | 5.86824 | 17.3259 |
| K5T46_RS12370 | DMAPP dephosphorylation | NudJ | 1.35242 | 0.03286 | 5.09011 | 6.88396 |
| K5T46_RS15840 | Dimethylallyltranstransferase | ispA | 1.6546 | 0.0369 | 12.7528 | 21.1009 |
| TcCDS | Chrysanthemyl diphosphate synthase | CDS | 0.33933 | 0.04321 | 50596.3 | 17168.9 |


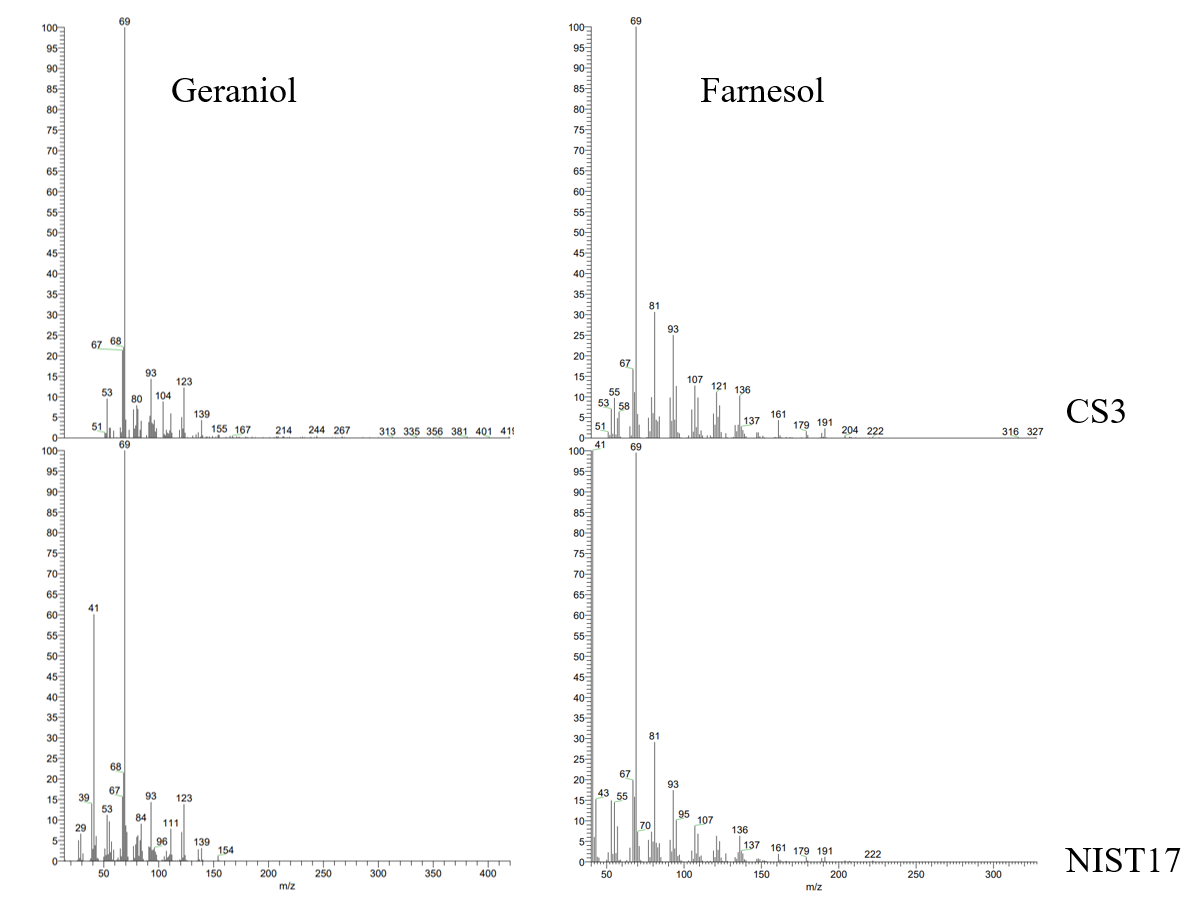


Fig. S1. Comparison of Geraniol and Farnesol mass spectrometry information with the NIST17 database (m/z: 50-500)


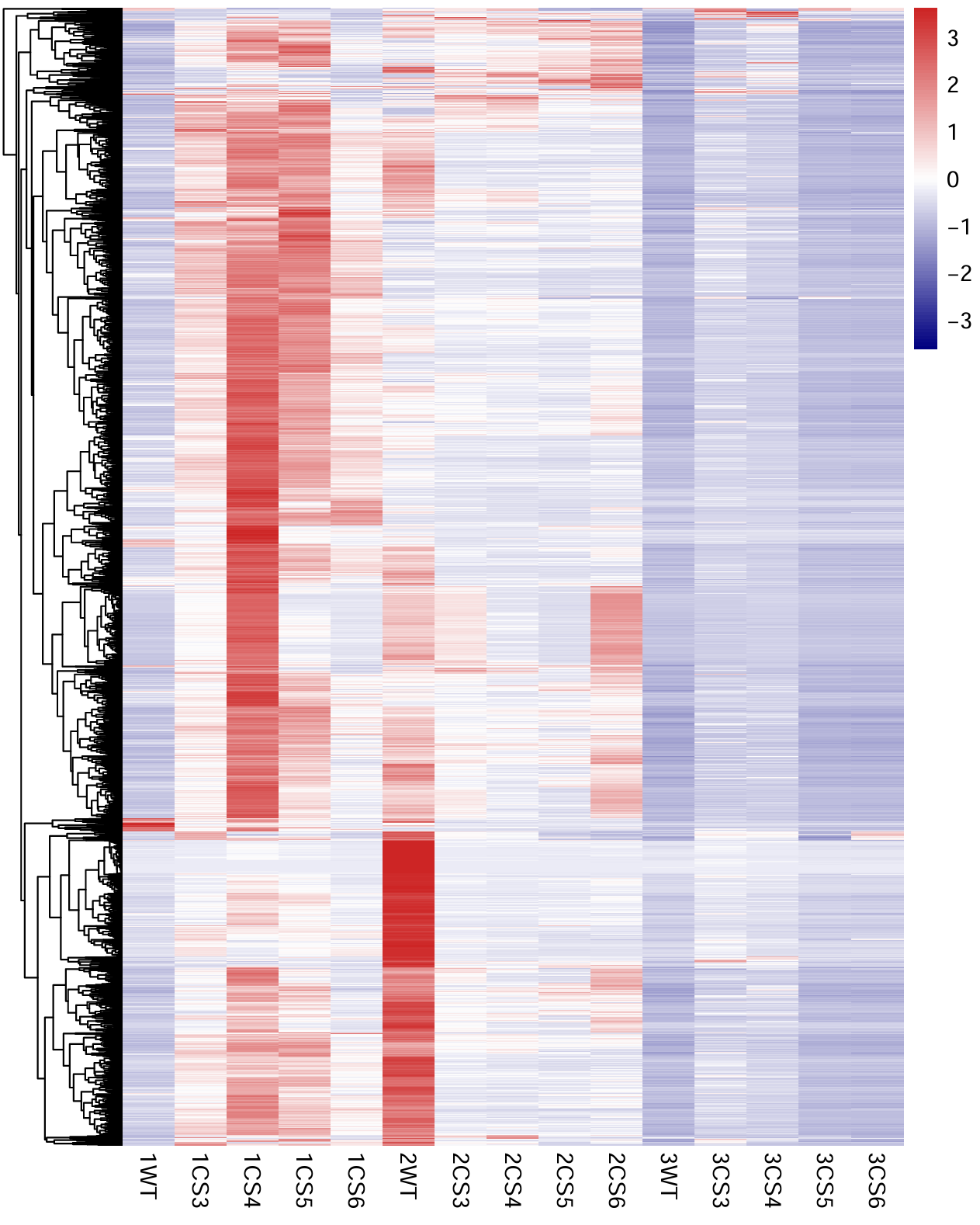


Fig. S2. Gene expression heat map.


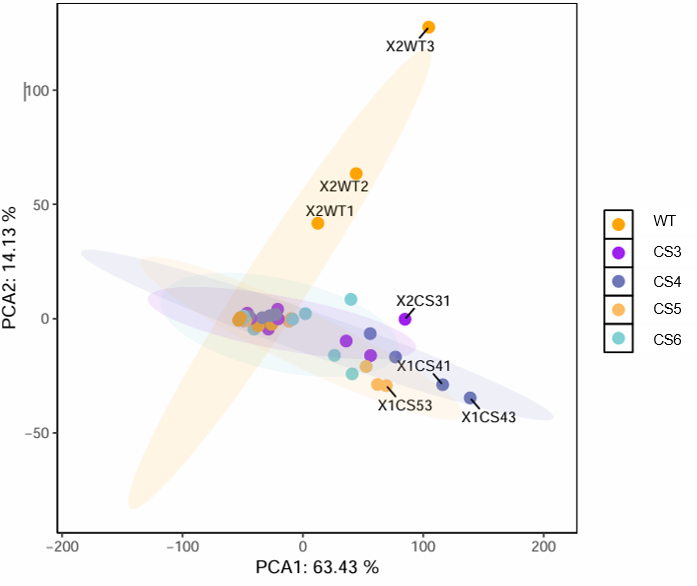


Fig. S3. PCA analysis based on strain transcriptome (N=3).


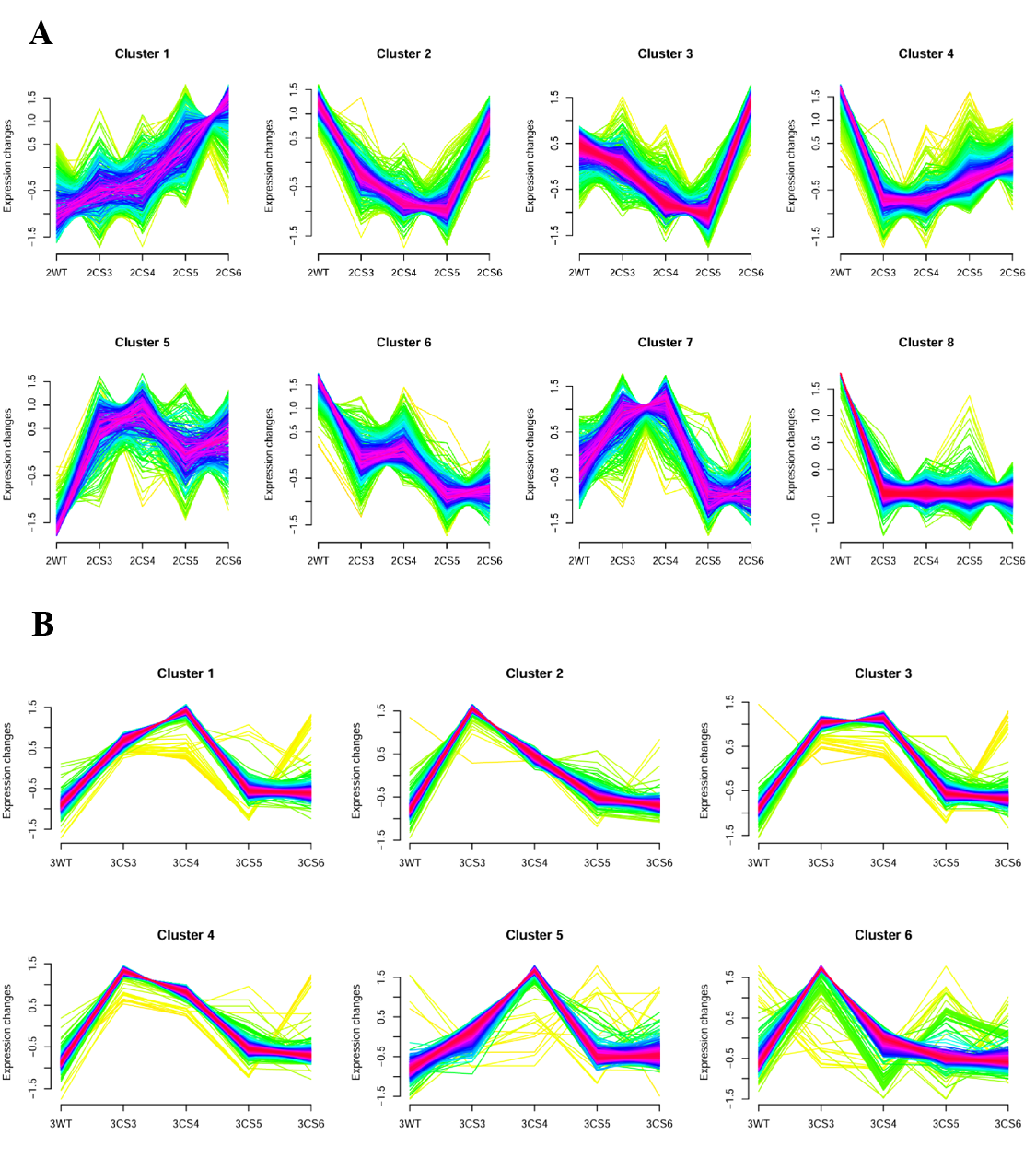


Fig. S4. Differential expression of genes between strains A. 16 hours after adding inducer. B. 48 hours after adding inducer.


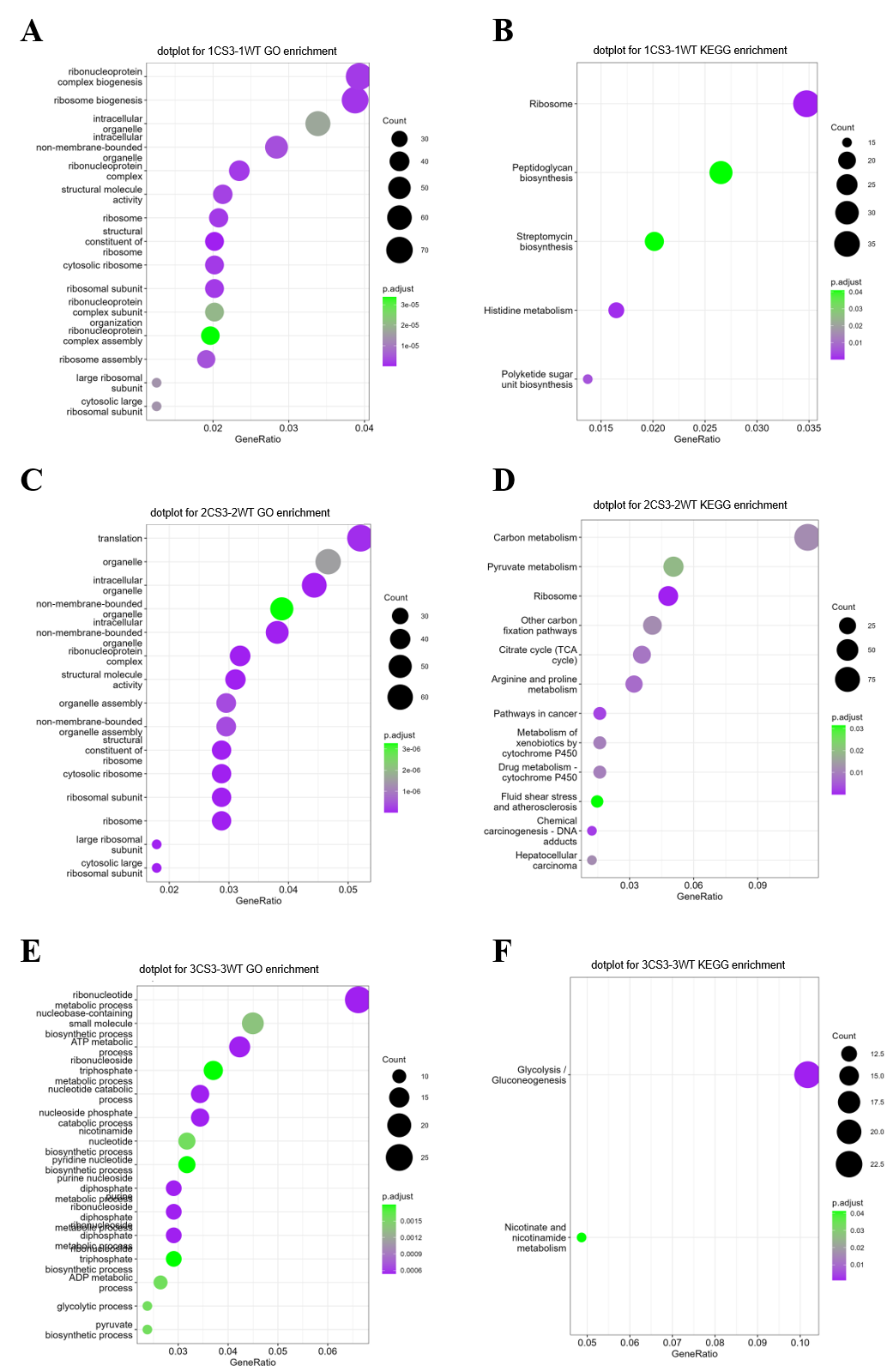


Fig. S5. GO enrichment and KEGG enrichment analysis of the transcriptome of CS3 strain relative to the wild type.


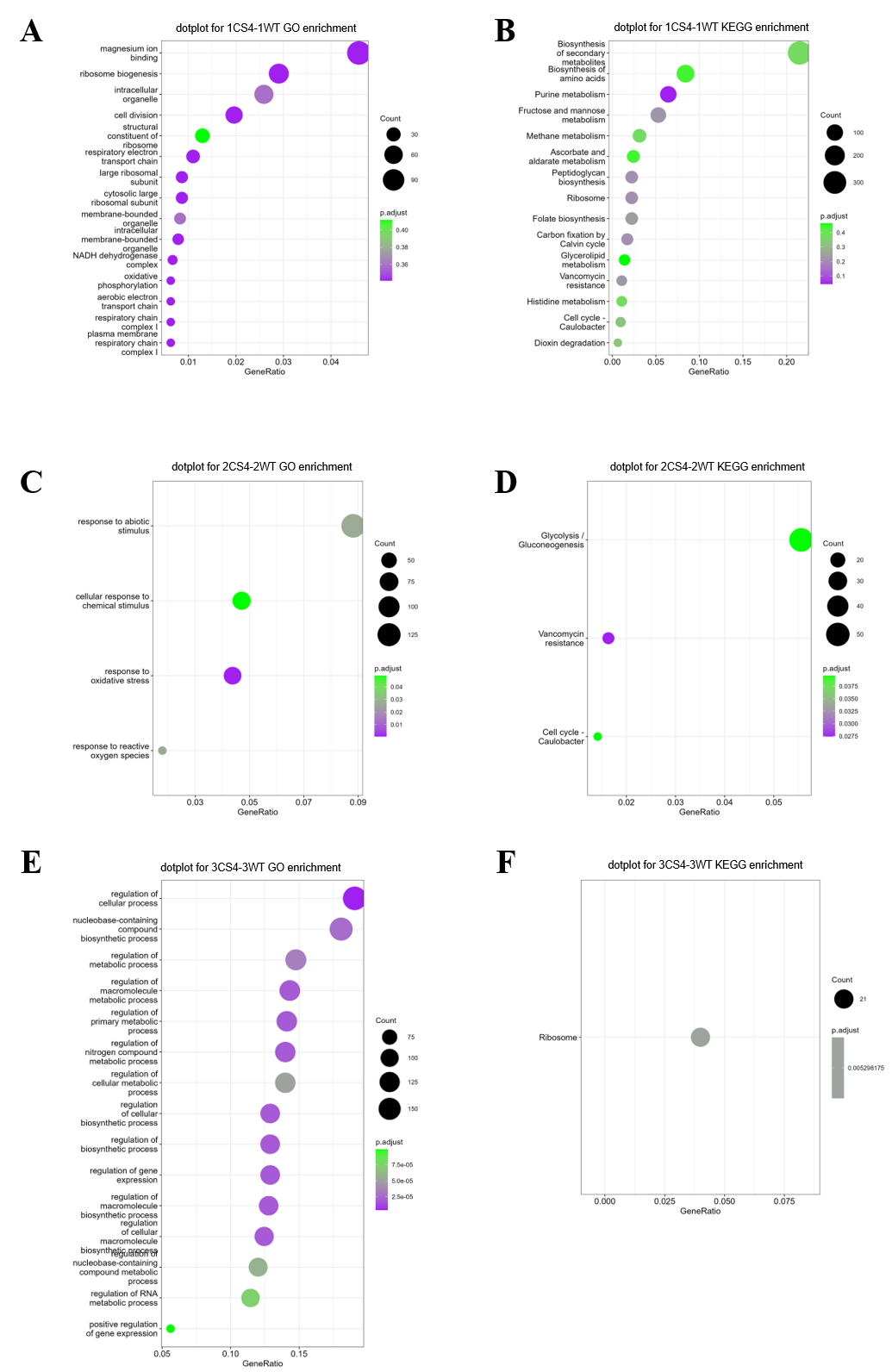


Fig. S6. GO enrichment and KEGG enrichment analysis of the transcriptome of CS4 strain relative to the wild type.


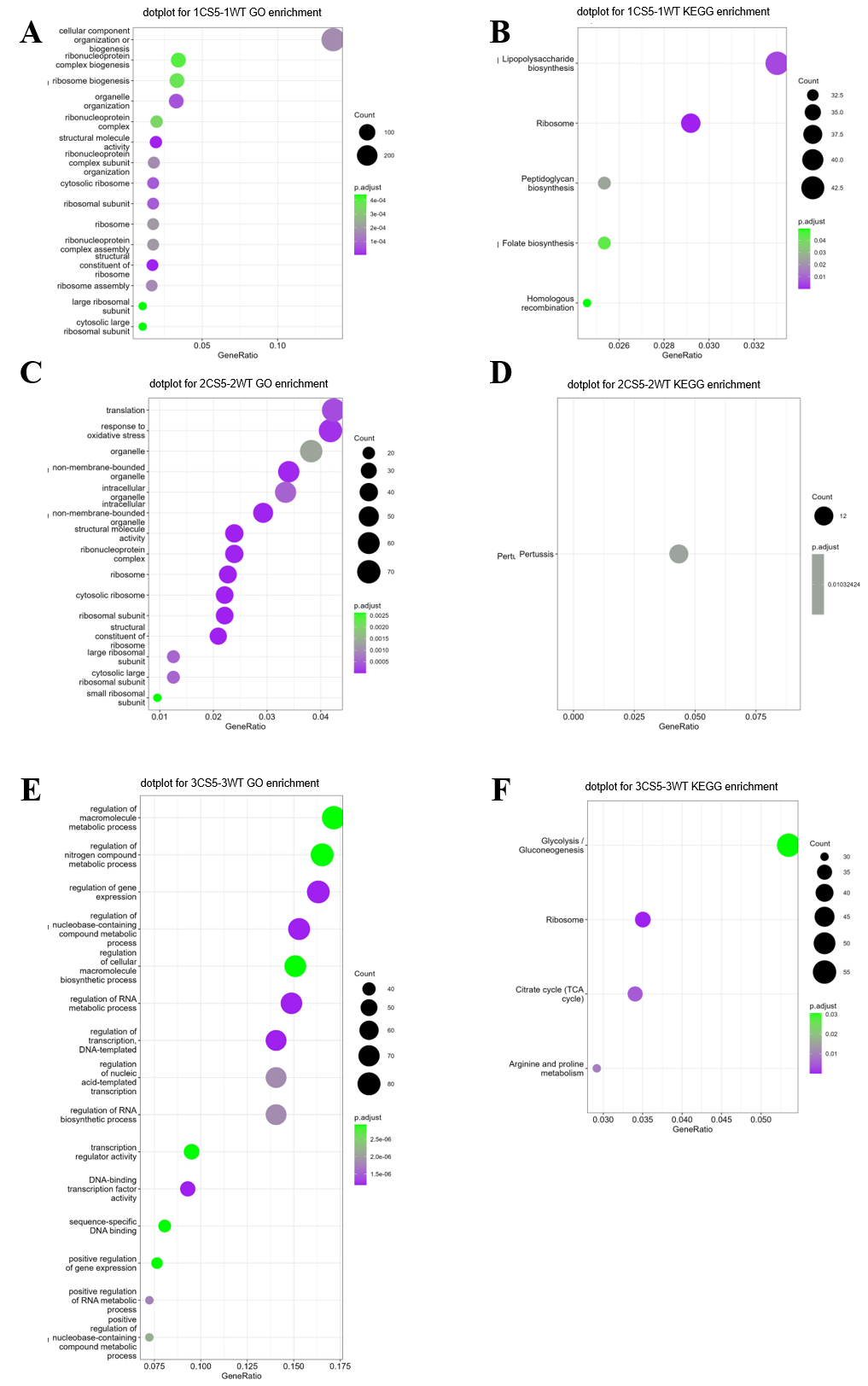
 Fig. S7. GO enrichment and KEGG enrichment analysis of the transcriptome of CS5strain relative to the wild type.


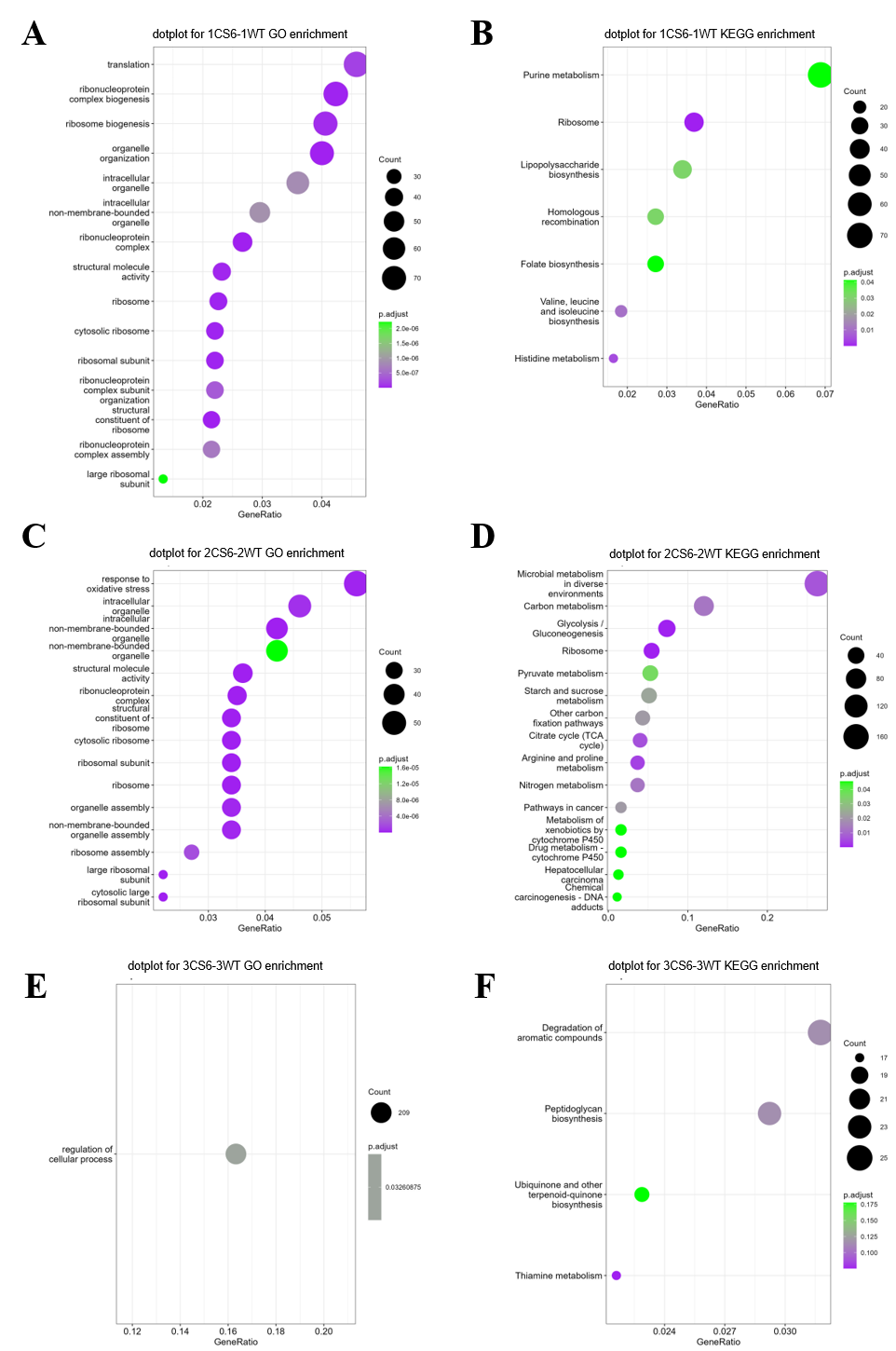


Fig. S8. GO enrichment and KEGG enrichment analysis of the transcriptome of CS6 strain relative to the wild type.


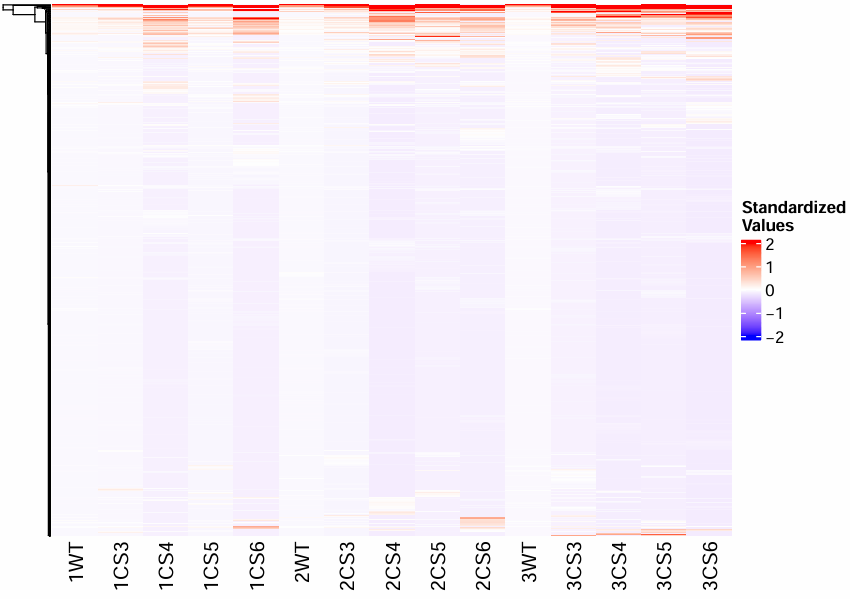


Fig. S9. Metabolite expression heat map.


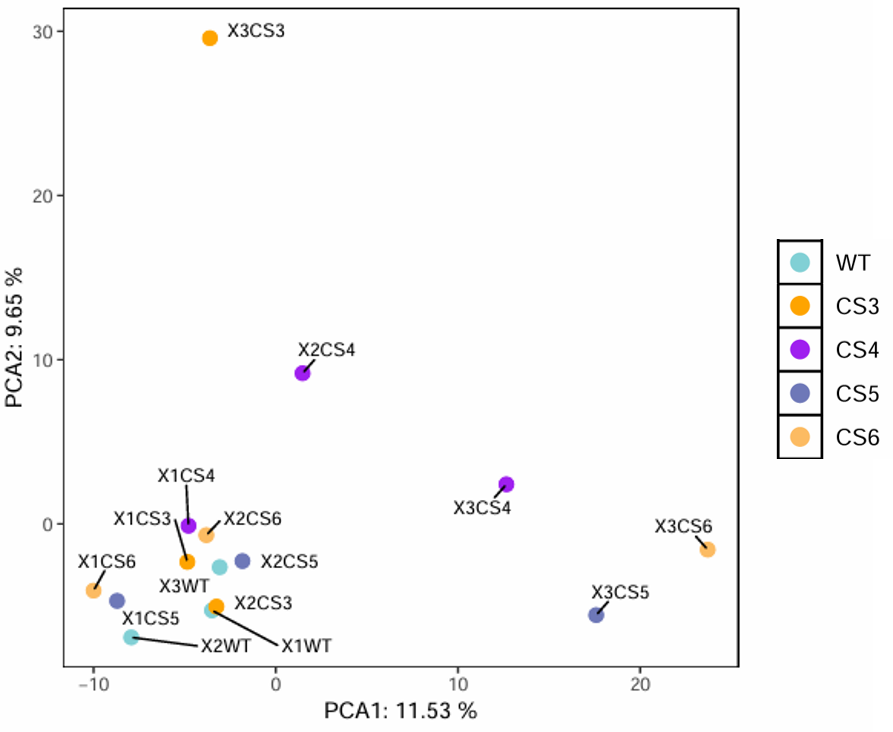


Fig. S10. PCA analysis based on strain metabolite expression (N = 3).


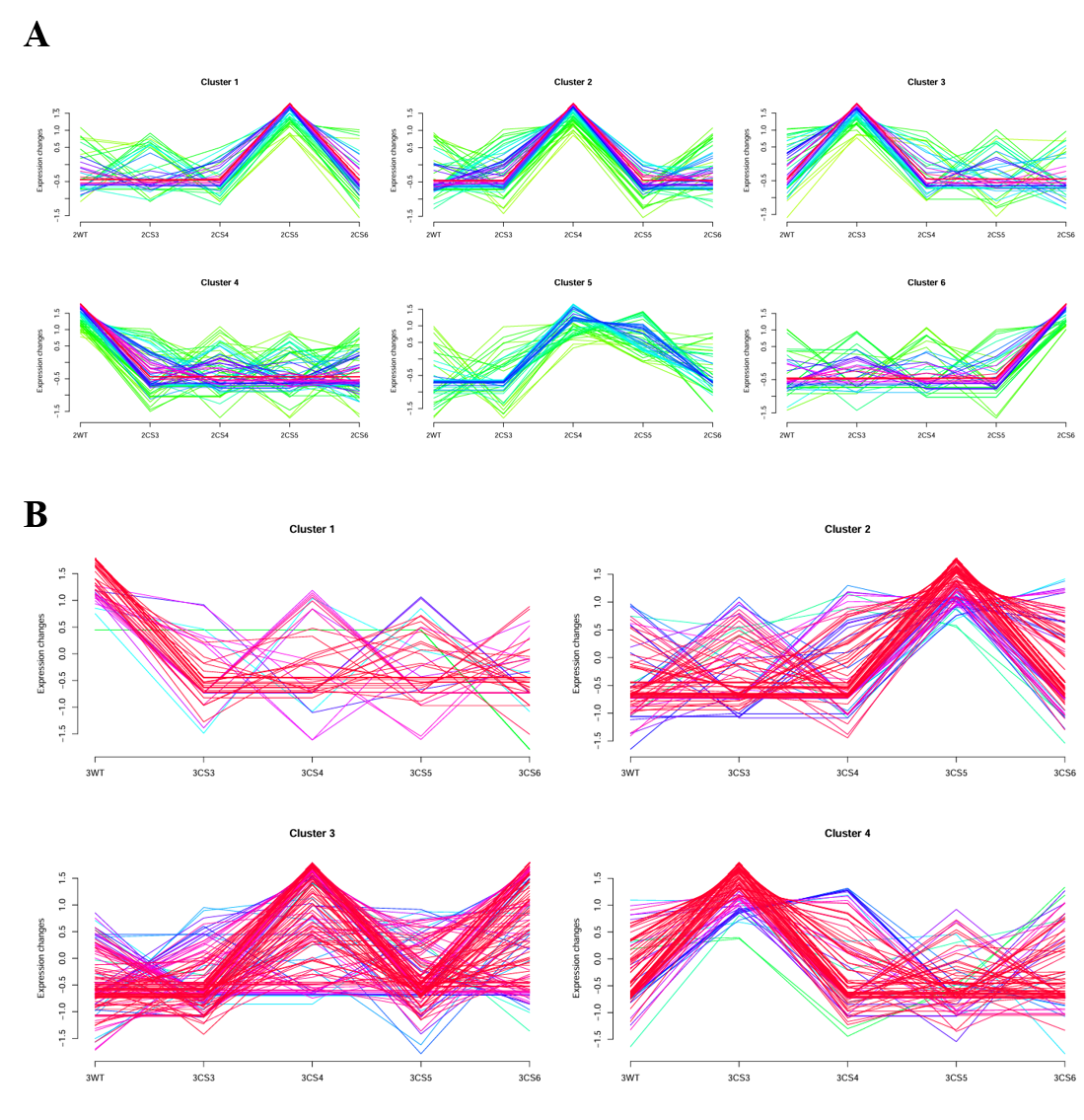


Fig. S11. Differential expression of metabolites in the strains. A. 16 hours after adding inducer. B. 48 hours after adding inducer.


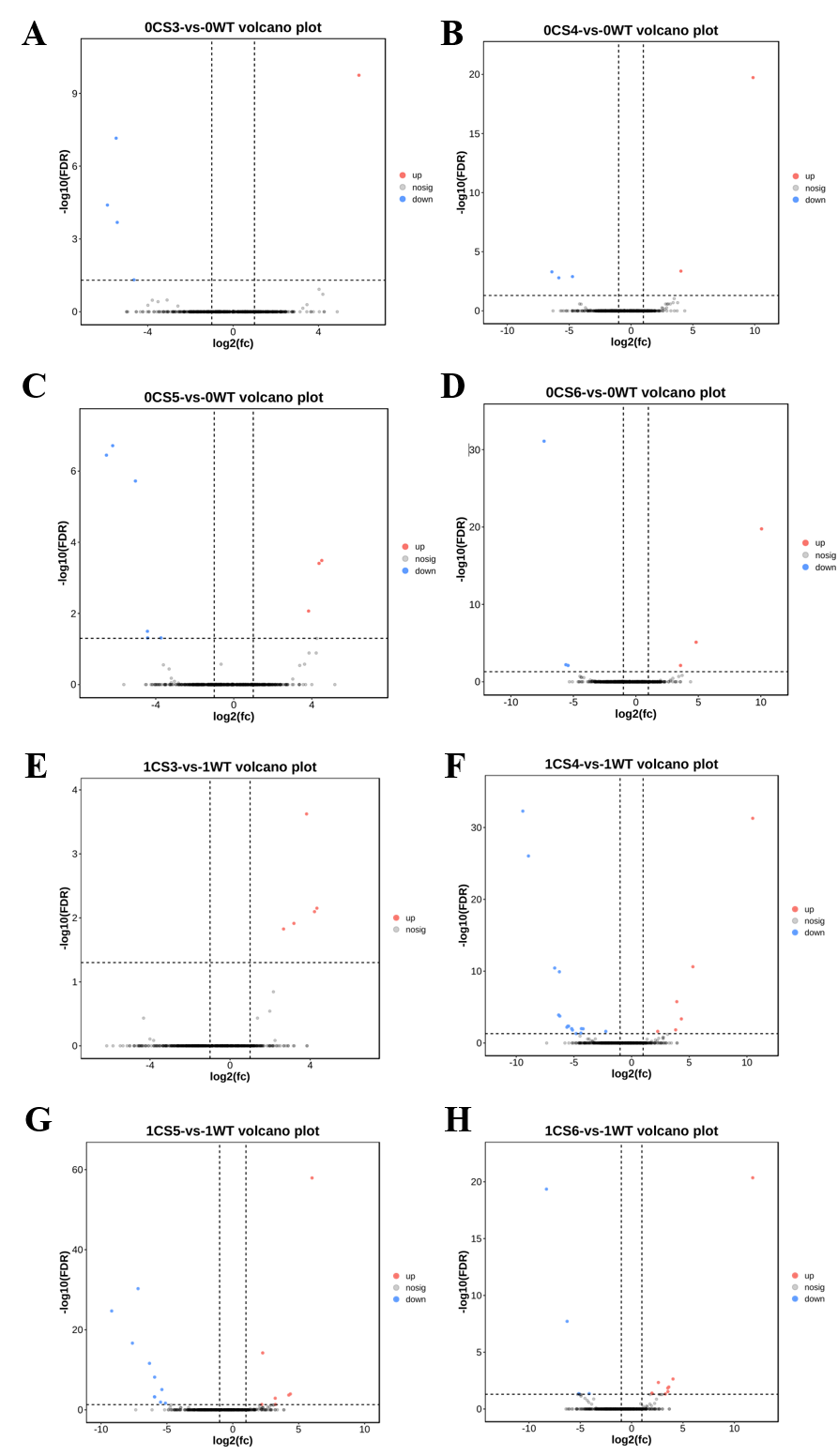


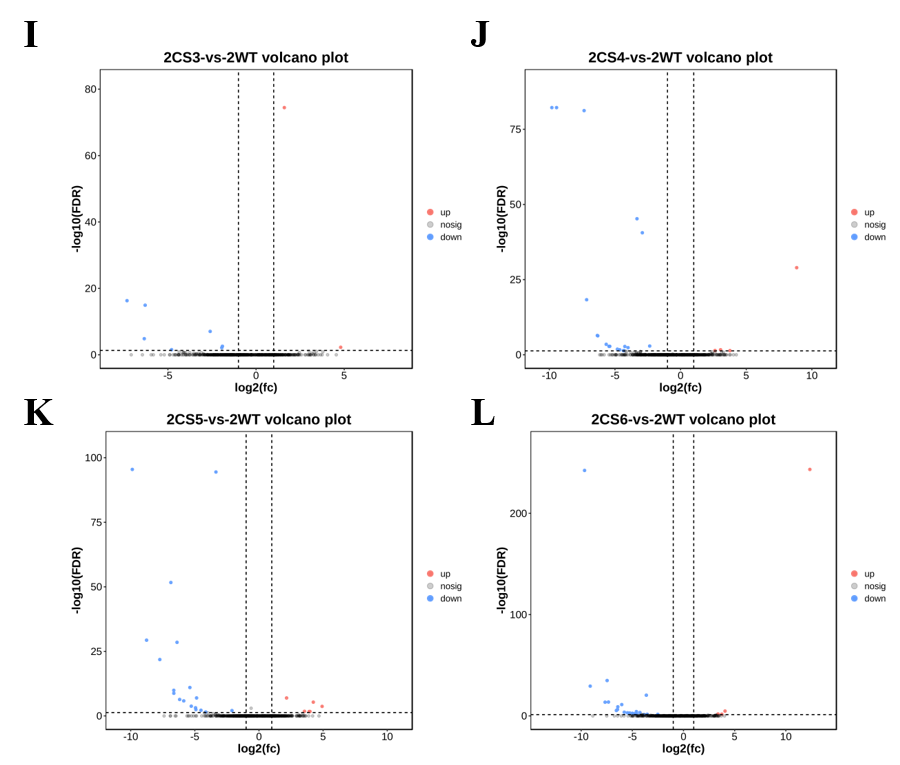


Fig. S12. Differences in gene metabolites at different stages and among different strains. A-D, initial metabolite changes induced by CS3, CS4, CS5, and CS6. E-H, changes in mid-term metabolites induced by CS3, CS4, CS5, and CS6. I-L, CS3, CS4, CS5, and CS6 induced changes in late metabolites. (N = 3)


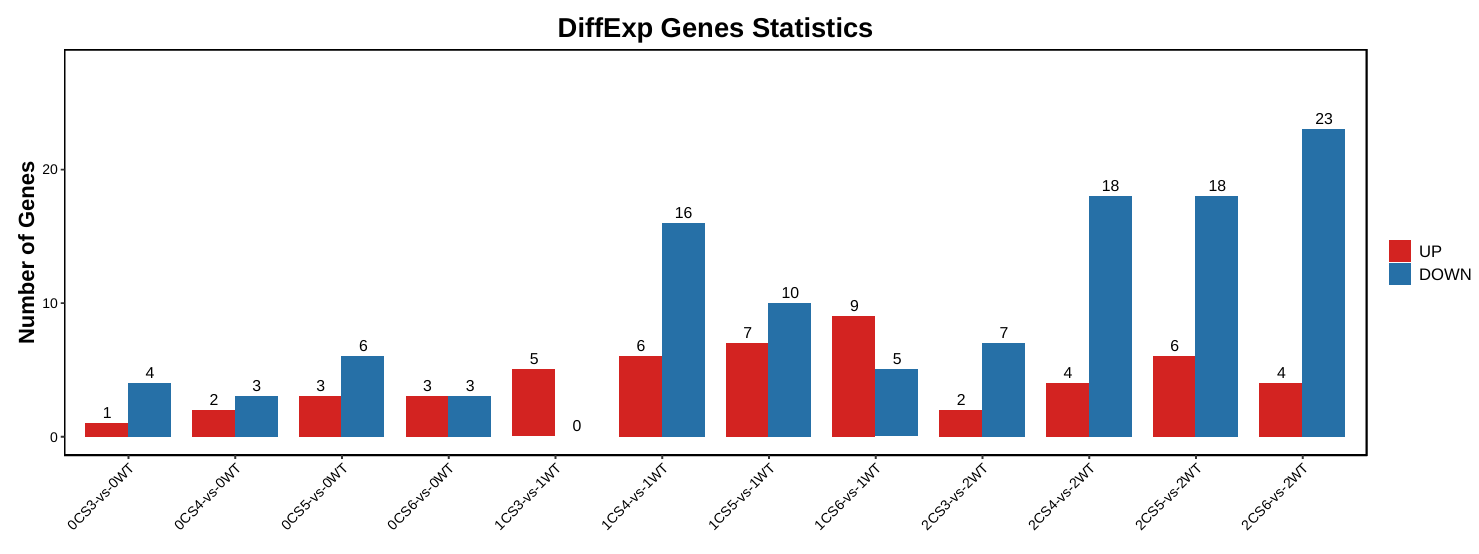


Fig. S13. Statistics of differential metabolites of CS3, CS4, CS5, and CS6 compared with the wild type (N = 3).


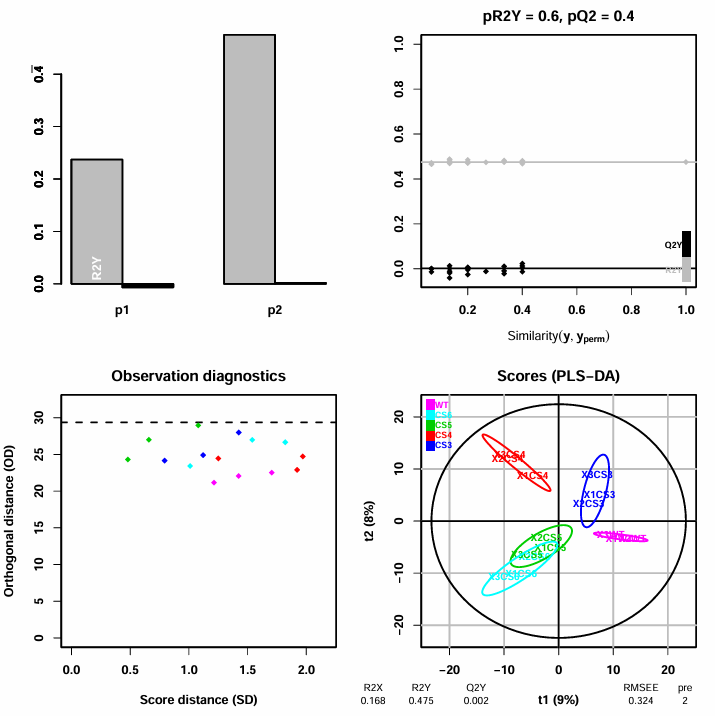


Fig. S14. OPLS-DA analysis of metabolome.


Fig. S15. Chrysanthemol titer of CS4 and CS5-1 strains within 48 hours


Fig. S16. The catalytic rate ratios of different enzymes interacting with the DMAPP.

Fig. S17. Changes in chrysanthemol titer under the simulation of ispA limiting flux 0.1-1.0.

Fig. S18. Compared to the CS5-1 model, the growth rate under different ispA gene expression levels were simulated in the CS5-3 model.

Fig. S19. Optimization of production conditions of chrysanthemol.

(A) Growth curve (yellow) and chrysanthemol production curve (green) of strain CS5-1. (B) Chrysanthemol titer of strain CS5-1 under different temperature conditions. (C) Chrysanthemol titer of strain CS5-1 in different culture media. (D) Growth curve (green) and chrysanthemol production curve (yellow) of strain CS5-1 at different IPTG inducer concentrations. (E) Comparison of chrysanthemol titer between strain CS5-1 and CS5-2 under baseline conditions. (F) Intracellular versus extracellular chrysanthemol concentrations in strain CS5-1. (G) Chrysanthemol titer of strain CS5-1 under optimized conditions (20°C, 2 × YT medium, 1.0 mM IPTG). N≥3.

Fig. S20. qPCR analysis of dehydrogenase gene copy number and *ispA* in optimized strains. A: Quantification of *ADH2* gene expression in CS6, CS7, and CS8 strains using qPCR (N = 3); B: Quantification of *ALDH1* gene expression in CS7, CS9, and CS10 strains using qPCR (N = 3); C: Quantification of *ispA* gene expression in CS10 and CS11 strains using qPCR (N = 3).

Fig. S21. qPCR analysis of dehydrogenase gene copy number in optimized strains. A: Quantification of *ADH2* gene expression in CS6, CS7, CS8 CS9 and CS10 strains using qPCR (N = 3); B: Quantification of *ALDH1* gene expression in CS7 and CS9 strains at different times using qPCR (N = 3).
